# Acyl Quinic Acid Derivatives Screened Out from *Carissa spinarum* by SOD-Affinity Ultrafiltration LC–MS and Their Antioxidative and Hepatoprotective Activities

**DOI:** 10.3390/antiox10081302

**Published:** 2021-08-18

**Authors:** Ye Liu, Felix Wambua Muema, Yong-Li Zhang, Ming-Quan Guo

**Affiliations:** 1 CAS Key Laboratory of Plant Germplasm Enhancement and Specialty Agriculture, Wuhan Botanical Garden, Chinese Academy of Sciences, Wuhan 430074, China; liuye@wbgcas.cn (Y.L.); fwambua83@mails.ucas.ac.cn (F.W.M.); zhangyongli@wbgcas.cn (Y.-L.Z.); 2 Sino-Africa Joint Research Center, Chinese Academy of Sciences, Wuhan 430074, China; 3 Innovation Academy for Drug Discovery and Development, Chinese Academy of Sciences, Shanghai 201203, China; 4 University of Chinese Academy of Sciences, Beijing 100049, China

**Keywords:** acyl quinic acid derivatives, antioxidant activity, hepatoprotective activity, UF–LC/MS screening, *Carissa spinarum*

## Abstract

*Carissa spinarum* Linn. has been utilized both in the food industry and as a traditional medicine for various ailments, while the responsible chemical components and action mechanisms of its antioxidative and hepatoprotective activities remain unclear. In this work, at least 17 quinic acid derivatives as potential ligands for the superoxide dismutase (SOD) enzyme from *Carissa* *spinarum* L. were screened out using the bio-affinity ultrafiltration with liquid chromatography mass spectrometry (UF–LC/MS), and 12 of them (**1**–**12**), including, three new ones (**1**–**3**), were further isolated by phytochemical methods and identified by high resolution electrospray ionization mass spectrometry (HR-ESI-MS) and extensive nuclear magnetic resonance (NMR) spectroscopic analysis. All of these isolated compounds were evaluated for their antioxidant activities by the 1,1-diphenyl-2-picrylhydrazyl (DPPH) and ferric-reducing antioxidant power (FRAP) methods. As a result, compounds **4** and **6**–**11** displayed similar or better antioxidant activities compared to vitamin C, which is in good agreement with the bio-affinity ultrafiltration with SOD enzyme. Then, these compounds, **4** and **6**–**11**, with better antioxidant activity were further explored to protect the L02 cells from H_2_O_2_-induced oxidative injury by reducing the reactive oxygen species (ROS) and Malondialdehyde (MDA) production and activating the SOD enzyme. To the best of our knowledge, this is the first report to use an efficient ultrafiltration approach with SOD for the rapid screening and identification of the SOD ligands directly from a complex crude extract of *Carissa spinarum*, and to reveal its corresponding active compounds with good antioxidative and hepatoprotective activities.

## 1. Introduction

*Carissa spinarum* Linn., which belongs to the family Apocynaceae and the genus *Carissa* [1,2], is extensively distributed in Africa, Asia, Australia, and various islands of the Indian Ocean [3]. *C. spinarum* has been used both in the food industry and as a traditional medicine for the treatment of various ailments [3]. Its raw fruits are used for making pickles, and ripe fruits for syrup and jelly production, and in Australia, these edible berries are known as native currants [3,4]. Traditionally, the leaves and roots of this plant have been extensively used for the treatment of malaria, diarrhea, rheumatism, snake bites, gastric ulcers, diabetes, and so on [2,3,5]. Modern research has also verified its various pharmacological potentials on anti-oxidation [6], anti-inflammation [7], anti-nociception [8], anti-bacterium [6], hepatic protection [9], and wound healing [10]. Phytochemical studies have been performed on the roots, stems, and leaves of *C. spinarum*, leading to the isolation of a total of 15 compounds [2], including sterols [11], lignans [12], flavonoids [13], cardiac glycosides [14], sesquiterpenes [12], and phenolic acids [15,16]. Only five compounds, consisting of two sterols [11], two cardiac glycosides [14], and one phenolic acids [15], have been identified from the roots of *C. spinarum* so far, which is not enough to elucidate its various pharmacological potentials. Our previous study revealed that ethyl acetate (EA) extracts from the root barks of *C. spinarum* exhibit promising antioxidant activity [17], while the responsible components and mechanism remain unexplored. 

At present, an integrative strategy combining bio-affinity ultrafiltration of target enzymes of interest with LC/MS (UF–LC/MS) has been developed and applied in the discovery of ligands for biological targets from complex samples, which allows the simultaneously high-throughput screening of the potential ligands for target enzymes and rapid identification of these candidate active molecules [18,19]. Inspired by the advantages of UF–LC/MS in the discovery of bioactive natural products and the rich experience of our research group in the application of this method coupled with various biological targets [20,21], the underlying components and mechanism of the antioxidant activity of an EA fraction of *C. spinarum* might be disclosed by UF–LC/MS coupled with related enzymes. Superoxide dismutase (SOD), a key endogenous antioxidant enzyme, is regarded as the main component of the defense system against oxidative stress caused by reactive oxygen species (ROS), which has been proven to be related to many physiological dysfunctions, such as cancer, hepatotoxity, and neurodegeneration [22]. Thus, in the present study, in order to explore the efficacious material basis of the antioxidant activity of *C. spinarum*, UF–LC/MS analysis was used to obtain SOD-combining compounds from *C. spinarum*, and 17 quinic acid derivatives were screened out. Then, for further structural identification and verification of the results above, UF–LC/MS-guided phytochemical isolation was performed, and 12 acyl quinic acid derivatives, including three new ones, were obtained. Finally, in vitro antioxidant and hepatoprotective assays were carried out to verify the antioxidant, ROS scavenging, and H_2_O_2_-induced hepatic protection abilities of the potential active compounds screened out. To the best of our knowledge, this is the first time UF–LC/MS method coupled with SOD has been employed in the phytochemical analysis of *C. spinarum*, and all these obtained compounds were first isolated from the genus *Carissa*. More importantly, this strategy also firstly succeeded in revealing the corresponding active compounds with good antioxidative and hepatoprotective activities directly from a complex crude extract of *C. spinarum*, and can provide a good showcased study for other natural products of interests.

## 2. Materials and Methods

### 2.1. Apparatus and Reagents

Thin layer chromatography (TLC) analyses were carried out on silica gel GF_254_ plates (Yantai Institute of Chemical Technology, Yantai, China), observed under ultraviolet (UV) light at 254 nm and sprayed with 10% H_2_SO_4_−EtOH, followed by heating on a hot plate. Medium pressure liquid chromatography (MPLC) was performed using Tauto TBP 5010 (Shanghai Tauto Biotech Co., Ltd., Shanghai, China). Column silica gel (200–300 mesh; Qingdao Marine Chemical Inc., Qingdao, China), Sephadex LH-20 gel (GE Healthcare, Chicago, USA), and ODS gel (S-50 µm, YMC, Tokyo, Japan) were used for column chromatography. A Smuwei C18 column (10 µm, 250 mm × 30 mm i.d.) on Hanbon NP7000 system (Hanbon Sci. and Tech., Jiangsu, China) was used for reversed-phase preparative high performance liquid chromatography (HPLC), a YMC-Pack ODS-A C18 (5 µm, 250 mm × 10 mm i.d.; YMC, Tokyo, Japan) column on the Agilent 1100 system (Agilent, Santa Clara, CA, USA) was used for reversed-phase semi-preparative HPLC, and a Waters Sunfire C18 (3.5 µm, 150 mm × 4.6 mm i.d.; Waters, Milford, CT, USA) column on the Agilent 1220 system (Aglient, Santa Clara, CA, USA) was applied for analytical HPLC. High resolution electrospray ionization mass spectrometry (HR-ESI–MS) data were acquired on an Agilent 1290 infinity II 6530C Q-TOF mass spectrometer (Agilent, Santa Clara, CA, USA) using a Waters ACQUITY UPLC BEH C18 column (1.7 µm, 50 mm × 2.1 mm i.d.; Milford, MA, USA). Optical rotations were measured on a PerkinElmer 341 polarimeter (PerkinElmer, Waltham, MA, USA). UV spectra were carried out on a PerkinElmer Lambda 35 UV/vis spectrometer (PerkinElmer, Waltham, MA, USA). 1D and 2D NMR spectra (^1^H–^1^H COSY: ^1^H–^1^H correlation spectroscopy; HSQC, heteronuclear single quantum correlation; HMBC, heteronuclear multiple bond correlation; NOESY, nuclear Overhauser effect spectroscopy) were recorded on a Bruker Avance 600 MHz NMR spectrometer (Bruker, Karlsruhe, Germany). Chemical shifts (*δ*) are expressed in parts per million (ppm) and coupling constants are given in Hertz (Hz). DPPH, FRAP, Sulforhodamine B (SRB), and ROS quantitation assays were all performed on a Tecan Infinite M200 PRO multi-functional microplate reader (Tecan, Männedorf, Switzerland).

### 2.2. Plant Material

The root barks of *C. spinarum* were collected from Mount Kenya of Meru, Kenya, and authenticated by a senior taxonomist, Professor Guangwan Hu, from the Key Laboratory of Plant Germplasm Enhancement and Specialty Agriculture of Wuhan Botanical Garden, Chinese Academy of Sciences, Wuhan, People’s Republic of China, where the voucher specimen (No. 20190501) has been deposited.

### 2.3. Extraction

The air-dried root barks of *C. spinarum* (3.2 kg) were ground and extracted with methanol (5.0 L × 4 times) by the ultrasonic technique (30 min × 2 per time) at room temperature. The concentrated crude extract (360.7 g) was suspended in H_2_O (3.0 L) and then sequentially partitioned with petroleum ether (3.0 L, 3 times), dichloromethane (CH_2_Cl_2_, 3.0 L, 3 times), ethyl acetate (EtOAc, 3.0 L, 3 times), and *n*-BuOH (3.0 L, 3 times).

### 2.4. Screening for Potential SOD Ligands by Ultrafiltration

The ultrafiltration screening procedure followed a previously reported method [18,20,21]. Briefly, 100 µL of the EtOAc fraction sample solution (5.0 mg/mL in Tris-HCl, pH 8.0) was incubated with active SOD (10 U) and inactivated SOD enzyme (10 U, boiled in a water bath for 15 min) at 37 °C. Thirty minutes later, the mixture was ultra-filtrated through 30 KD ultrafiltration membranes (Millipore, 0.5 mL) by centrifuging at 8000 rpm for 10 min, and the membranes were then washed by 200 µL of Tris-HCl (pH 8.0) three times and the filtrates were discarded. Subsequently, 200 µL of MeOH was added into the ultrafiltration tubes, allowed to stand for 10 min, followed by centrifugation at 8000 rpm for 10 min, and this process was repeated three times. The filtrates were collected and freeze-dried by a lyophilizer, and were then dissolved in 50 µL of MeOH and analyzed by HPLC–UV and HPLC–ESI–MS/MS. The binding degrees between the molecule ligands and SOD were assessed by the enrichment factor (EF) values, which can be calculated by the following equation: EF(%) = (A_s_ − A_c_)/A_0_, where A_s_, A_c_, and A_0_ represent the HPLC peak areas obtained from incubation of the *C. spinarum* EA fraction with activated, inactivated, and no SOD, respectively. Additionally, all of the compounds were identified by comparison with the corresponding isolated pure compounds or the reported literature.

### 2.5. HPLC–UV and HPLC–ESI–MS/MS Analyses

The HPLC–UV analysis was performed on an Agilent 1220 system with a Waters Sunfire C18 (3.5 µm, 150 × 4.6 mm i.d.; Waters, Milford, CT, USA) column eluted by the following procedure: 0–15 min, 5–20% ACN−H_2_O; 15–45 min, 20–45% ACN−H_2_O (the water was supplied with 0.1% formic acid). The UV spectra were detected at 280 nm with an injection volume of 10 µL and a flow rate 0.8 mL/min. The HPLC–ESI–MS/MS data were collected on a Thermo Accela 600 series HPLC connected to a TSQ Quantum Access MAX mass spectrometer (Thermo Fisher Scientific, Waltham, MA, USA) in a negative ion mode; the detailed condition is as follows: Spray voltage of 3000 V; collision energy of 40 V; vaporizer temperature of 350 °C; capillary temperature of 250 °C; mass range of 100–1000; sheath gas pressure of 40 psi; aux gas pressure of 10 psi. 

### 2.6. Isolation

The EtOAc fraction (14.0 g) was subjected to MPLC (ODS C18, 50 µm, 180.0 g), and eluted with a mixture of MeOH and H_2_O gradient system from 50% to 90% to afford five fractions, A–E. Fraction A was chromatographed over MPLC with an ODS C18 column eluted with the above system from 20% to 55% MeOH−H_2_O to afford subfractions, A_1_−A_4_. Fraction A_1_ was subjected to preparative HPLC (250 mm × 30 mm i.d., C18 10 µm, flow rate: 10.0 mL/min) performed with an elution program of 15%−65% MeOH−H_2_O in 35 min to afford subfractions A_11_−A_14_. A_12_ was further purified by semi-preparative RP-HPLC (MeOH−H_2_O−HCOOH, 15:85:0.05, 250 mm × 10 mm i.d., C18 5 µm, flow rate: 2.0 mL/min) to afford compounds **1** (2.4 mg), **2** (6.8 mg), and **4** (4.8 mg). A_13_ was subjected to semi-preparative RP-HPLC, performed with MeOH−H_2_O−HCOOH (21:79:0.05) to yield compound **5** (8.8 mg). Fraction A_2_ was subjected to preparative HPLC (flow rate: 10.0 mL/min, 20–65% MeOH−H_2_O in 38 min) to obtain subfractions A_21_−A_23_. Then, A_22_ was purified by semi-preparative RP-HPLC performed with an elution program of 20–30% MeOH−H_2_O with 0.05% formic acid in 30 min to afford compounds **3** (1.4 mg), **6** (27.9 mg), and **12** (2.0 mg), and A_23_ was later separated by semi-preparative RP-HPLC in gradient 15–25% ACN−H_2_O with 0.05% formic acid in 35 min to obtain compounds **7** (47.0 mg), **9** (9.3 mg), and **10** (11.2 mg). Fraction A_3_ was successively purified by preparative HPLC (flow rate: 10.0 mL/min, 20–70% MeOH−H_2_O in 40 min) and semi-preparative RP-HPLC (ACN−H_2_O−HCOOH, 15:85:0.05, flow rate: 2.0 mL/min) to yield compounds **8** (14.3 mg) and **11** (5.1 mg).

*Data for 3-O-vanilloylquinic acid (**1**)*: Colorless oil; [α${]}_{D}^{20}$–7.5 (*c* 0.12, MeOH); UV (MeOH, Figure S9) *λ*_max_ (log *ε*) 204 (4.72), 218 (4.69), 262 (4.47), 291 (4.22) nm; ^1^H NMR (CD_3_OD, 600 MHz), ^13^C NMR (CD_3_OD, 150 MHz) and 2D NMR data, see Figures S2–S8; (–)-HRESIMS [M–H]^–^ *m/z* 341.0883 (calculated for C_15_H_17_O_9_, 341.0873, *∆* +2.9 ppm, Figure S1).

*Data for 3-O-syringoylquinic acid (**2**)*: Colorless oil; [α${]}_{D}^{20}$–4.8 (*c* 0.23, MeOH); UV (MeOH, Figure S18) *λ*_max_ (log *ε*) 215 (4.60), 274 (4.23) nm; ^1^H NMR (CD_3_OD, 600 MHz),^13^C NMR (CD_3_OD, 150 MHz) and 2D NMR data, see Figures S11–S17; (–)-HRESIMS [M–H]^–^ *m/z* 371.0984 (calculated for C_16_H_19_O_10_, 371.0978, *∆* +1.6 ppm, Figure S10).

*Data for 3,4-Di-O-syringoylquinic acid (**3**)*: Colorless oil; [α${]}_{D}^{20}$–25.7 (*c* 0.07, MeOH); UV (MeOH, Figure S27) *λ*_max_ (log *ε*) 210 (4.59), 264 (4.04) nm; ^1^H NMR (CD_3_OD, 600 MHz), ^13^C NMR (CD_3_OD, 150 MHz) and 2D NMR data, see Figures S20–S26; (–)-HRESIMS [M–H]^–^ *m/z* 551.1401 (calculated for C_25_H_27_O_14_, 551.1401, *∆* 0 ppm, Figure S19).

### 2.7. DPPH Radical Scavenging Activity Assay

The DPPH radical scavenging activity screening of the isolates was based on a method reported previously [23]. First, 190 µL of the DPPH solution (100 µM) and 10 µL of the tested samples with various concentrations were placed in a 96-well plate and then incubated for 30 min in darkness at room temperature. Vitamin C was used as the positive control and methanol as the blank control. After the incubation, the absorbance of the reaction mixture was measured with a microplate reader at 517 nm, and the IC_50_ value was calculated by GraphPad Prism software. Following the same procedures above, all of the tests were repeated three times independently.

### 2.8. Ferric Reducing Antioxidant Power (FRAP) Assay

A FRAP assay was performed following a previously reported method with modifications [24]. Briefly, 190 µL of freshly prepared FRAP working solution, which was composed of 300 mM of acetate buffer (pH 3.6), 20 mM of FeCl_3_·6H_2_O solution, and 10 mM of TPTZ solution at a ratio of 10:1:1 (*v*/*v*/*v*), was pre-warmed at 37 °C, and 10 µL of the sample solutions of various concentrations were mixed in a 96-well plate and incubated for 10 min at 37 °C. Then, the absorbance of the mixture was monitored by a microplate reader at 593 nm. A standard curve was established by FeSO_4_·7H_2_O, and the total antioxidant capacities of these tested compounds were calculated accordingly and expressed in terms of the FeSO_4_ values. Vitamin C was used as a positive control, and all of the tests were repeated three times independently. 

### 2.9. Hepatoprotective Activity Assay

The hepatoprotective activities of the compounds of interest were evaluated according to a published method with minor modifications [25]. The L02 cell line was maintained in RPMI-1640 medium (Hyclone, UT, USA) with a 10% fetal bovine serum and 1% penicillin (100 U/mL)–streptomycin (100 µg/mL) mixture solution (Hyclone, UT, USA) in a 37 °C humidified incubator supplied with 5% CO_2_. Cells in the logarithmic growth phase were seeded in 96-well plates (2 × 10^4^ cells in 120 μL of culture medium per well); after 24 h of incubation, the cells were pretreated with 15 μL of the solution of the tested compounds with final gradient concentrations (25, 5, 1 μM) in triplicate for 12 h. The model and normal control groups were added to with equivalent amounts of PBS, and vitamin C were used as the positive control. Then, 15 μL of a H_2_O_2_ solution with a final concentration of 300 μM was added and incubated for 4 h (equivalent PBS for the normal control group). Finally, the cell viability was evaluated using a sulforhodamine B (SRB) test kit (BestBio, Shanghai, China) according to the manufacturer’s instructions. 

### 2.10. Quantitation and Photography of ROS in the Hepatoprotective Assay

ROS detection was performed by 2′, 7′-Dichlorodihydrofluorescin diacetate (DCFH-DA) based fluorescent method following the instructions of a commercial ROS assay kit (Beyotime, Shanghai, China). The L02 cell pretreatment was the same as that in the hepatoprotective activity assay procedure. Following the treatment of H_2_O_2_, DCFH-DA diluted with RPMI-1640 medium (final concentration of 10 µM) was added and maintained in the incubator for 30 min, and then the cells were washed thrice with PBS and photographed by a cell imaging multi-mode reader (Cytation 1, BioTek, Winooski, VT, USA). Lastly, the fluorescence intensity was recorded on a microplate reader (*λ*_ex_ = 488 nm; *λ*_em_ = 525 nm) and the fluorescence values were calibrated by the corresponding cell viabilities using the SRB method. In the same way, all of the tests were repeated three times.

### 2.11. Biochemical Assays

The determinations of the MDA (malondialdehyde) level and the SOD (super oxide dismutase) enzyme activity were measured by corresponding commercial kits purchased from Nanjing Jiancheng Bioengineering Institute (Nanjing, China) following the manufacturer’s instructions. The L02 cells were seeded in 6-well plates and treated by the same procedure as the hepatoprotective activity assay, and the culture supernatant and cells were then collected separately. Then, 200 µL of the culture supernatant of each group was used for the MDA level assays (thibabituric acid method (TBA)). The cells were disrupted by repeated freezing and thawing, and 20 µL of the suspension (after quantify and dilution) of each group were was for the SOD activity assays (Water Soluble Tetrazolium (WST-1) method). The group setting, reagents used, and data analyses were guided by the kit instructions. 

### 2.12. Statistical Analyses

The activity data in this work are expressed as means ± standard deviation (SD). The standard curves used in the FRAP assays were generated by Microsoft Excel (Microsoft Corporation, Redmond, WA, USA). The IC_50_ values were calculated by GraphPad Prism 5 (GraphPad Software Inc., San Diego, CA, USA) using the log (inhibitor) vs. normalized response–variable slope method. The statistical analyses in this work were performed by GraphPad Prism 5 (GraphPad Software Inc., San Diego, CA, USA) using one-way ANOVA Newman–Keuls multiple comparison tests.

## 3. Results and Discussion

### 3.1. Screening for Potential SOD Ligands by UF–LC/MS

**Figure 1:**
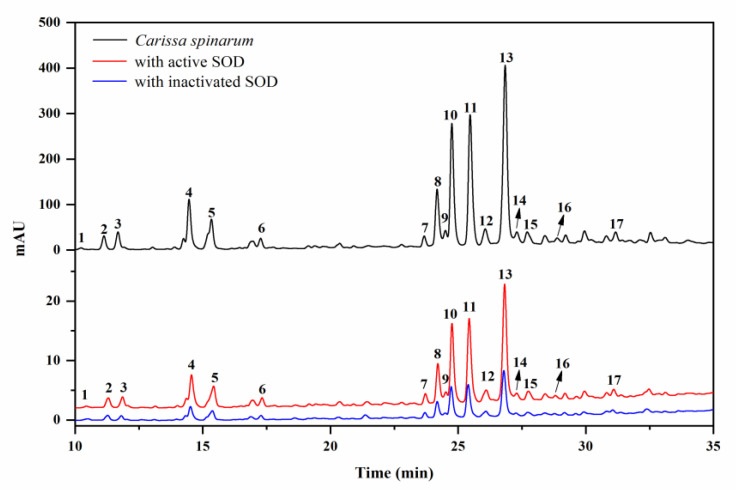
HPLC chromatograms of the *Carissa spinarum* ethyl acetate (EA) fraction without ultrafiltration (black line, top), ultrafiltration with activated superoxide dismutase (SOD) (red line, middle), and ultrafiltration with inactivated SOD (blue line, bottom).

Considering the key role SOD plays in the defense against oxidative stress, the SOD-based UF–LC/MS method was used to rapidly screen for the compounds in *C. spinarum* with good binding ability to SOD, which might be regarded as potential SOD ligands contributing to the antioxidant activity of the extract. As shown in Figure 1, the components released from the binding complex in the bio-affinity ultrafiltration screening were analyzed by HPLC, in which 17 peaks with various binding abilities were observed. Herein, the enrichment factor (EF) values were employed to express the binding degrees between the small molecule ligands and SOD [18]. Accordingly, based on the variations of the peak areas before and after incubation with activated or inactivated SOD enzymes, the EF values of the corresponding peaks are listed in Table 1. As shown in Table 1, peak 17 (compound **10**) possessed the highest binding degree (3.53%), followed by peak 15 (3.51%), peak 11 (compound **7**, 3.30%), peak 10 (compound **6**, 3.16%), and peak 8 (compound **12**, 3.16%), while peak 1 (compound **1**, −0.24%), followed by peak 2 (compound **2**, 2.34%), showed the lowest binding ability. As reported, the different competitive binding interactions between the bioactive compounds and SOD might account for the differences in the EF values [26].

Identification of these compounds with varying affinities to SOD was carried out by comparing their LC–MS/MS data and retention time with those reported data in the literature and the corresponding isolated pure compounds in this work. The retention time (Rt), quasi-molecular ion ([M–H]^–^ in negative ion mode), and characteristic fragments are listed in Table 1. Peaks 1–15 were all determined as quinic acid derivatives according to their coexisting typical ions at *m/z* 191, which belong to the [M–H]^–^ ion for quinic acid [27]. Among these compounds, peaks 3–5 were identified as caffeoylquinic acid isomers possessing the same quasi-molecular ion at *m/z* 353 and fragment ion at *m/z* 179 for the caffeoyl group, which were further distinguished by the retention times with the isolated standards and reference data [27]. Similarly, peaks 10, 11, and 13, with the same [M–H]^–^ ion at *m/z* 515, were determined to be dicaffeoylquinic acid isomers for the characteristic fragments at *m/z* 353, 335, and 191 [27], and these isomers were verified by comparison with the isolated corresponding pure compounds. Combined with the parent ion and coexisting fragment ion at *m/z* 197 attributed to the syringoyl group [28], peak 2 was determined as syringoylquinic acid, which was then isolated and proven to be a new compound according to the NMR data. Meanwhile, peaks 6, 7, and 12, exhibiting an [M–H]^–^ ion at *m/z* 551, were deduced as disyringoylquinic acid isomers, while only peak 6 was obtained and further verified as a new compound. Simultaneously, peak 8 was explicitly identified as 4-*O*-caffeoyl-3-*O*-syringoylquinic acid and confirmed by the retention time with the isolated standard compound, while peak 15 was tentatively identified as a caffeoyl–sinapoylquinic acid isomer. Based on the quasi-molecular ion at *m/z* 341 and the fragment ion at *m/z* 167 attributed to the vanilloyl group [28], peak 1 was likely vanilloyl substituted quinic acid, which was further elucidated as a new compound, 3-*O*-vanilloylquinic acid, by the isolated compound data. Accordingly, peaks 9 and 14 were determined as caffeoyl–vanilloylquinic acid isomers by the similar fragment ions. The *m/z* values of the [M–H]^–^ ion for peaks 16 and 17 were 14 Da more than for dicaffeoylquinic acid isomers, indicating the existence of a methyl substituent; therefore, these two peaks were determined unequivocally, as shown by the comparison of isolated pure compounds.

### 3.2. Structure Elucidation of the Isolated Compounds

The EA fraction was found to be rich in quinic acid derivatives with considerable bio-affinity to SOD, and was subjected to a series of chromatographic steps guided by the screening result to obtain 12 acyl quinic acids (**1**–**12**), which corresponded to the UF–LC/MS spectrum as shown in Table 1 and Figure 2. To the best of our knowledge, all of these compounds were first isolated from the genus *Carissa* [2], and compounds **1**–**3** are newly identified, as they have not been reported to date. Their structures were determined by comprehensive spectroscopic methods as shown in Figures S1–S27, and the detail structural elucidations of compounds **1**–**3** are discussed below.

**Figure 3:**
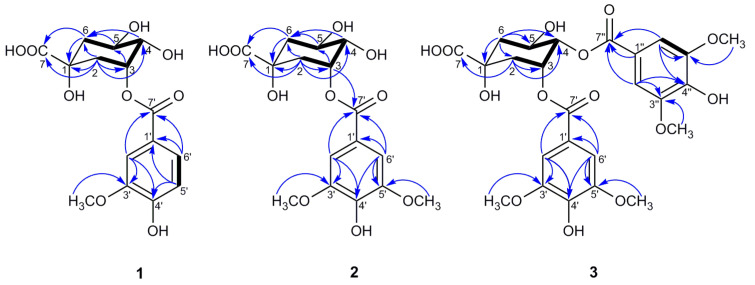
Key HMBC (→) and 1H–1H COSY (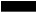) correlations of compounds **1**–**3**.

Compound **1** was isolated as a colorless oil. The negative HR-ESI–MS data showed an ion peak at *m/z* 341.0883 [M–H]^–^ (calculated for 341.0873), indicating its molecular formula of C_15_H_18_O_9_. The ^1^H NMR data (Table 2) of **1**, combined with the HSQC spectrum, displayed an ABX spin system attributed to a 1,3,4-trisubstituted benzene system (*δ*_H_ 7.67 (1H, d, *J* = 1.9 Hz, H-2′), 7.63 (1H, dd, *J* = 8.2, 1.9 Hz, H-6′), and 6.84 (1H, d, *J* = 8.2 Hz, H-5′)), which is suggestive of the existence of a vanilloyl moiety, along with the methoxy group signal at *δ*_H_ 3.91 (3H, s, 3′-OCH_3_) [28]. Additionally, the above inference was confirmed by the corresponding ^13^C NMR signals (Table 2) and the HMBC correlations of OCH_3_/C-3′; H-2′/C-3′, C-4′, C-7′; H-5′/C-1′, C-4′; and H-6′/C-1′, C-7′ (Figure 3). According to the ^1^H NMR (Table 2) and HSQC data, the remaining signals of three oxygenated methine protons at *δ*_H_ 5.50 (1H, q, *J* = 3.7 Hz, H-3), 4.21 (1H, td, *J* = 9.0, 4.0 Hz, H-5), and 3.70 (1H, dd, *J* = 8.4, 3.2 Hz, H-4), together with two methylene groups (*δ*_H_ 2.24 (1H, dd, *J* = 14.7, 3.6 Hz, H-2_ax_), 2.18 (1H, overlapped, H-2_eq_), 2.17 (1H, dd, *J* = 13.5, 3.6 Hz, H-6_eq_), and 1.97 (1H, dd, *J =* 13.5, 9.6 Hz, H-6_ax_)) were observed, which implies the presence of a quinic acid moiety [28,29,30]. The deduction above was further supported by the characteristic ^13^C NMR signals (Table 2) at *δ*_C_ 179.0, 75.6, 75.0, 68.5, 73.2, 41.5, and 37.0 and the HMBC correlations shown in Figure 3 [29,30]. Based on the above evidences, the structure of **1** should be a quinic acid substituted by a vanilloyl group.

**Figure 4:**
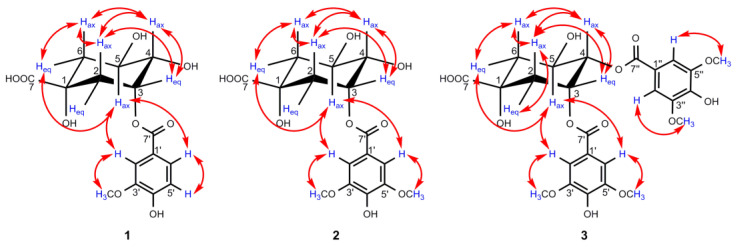
Key NOESY (↔) correlations of compounds **1**–**3**.

The ^1^H NMR signal of H-4 in the quinic acid unit was always well defined by the ^1^H−^1^H COSY cross peaks with both oxygenated methine protons H-3 and H-5 (Figure 3), while the assignments of H-2/H-3/H-5/H-6 could be approached by the analyses of all of the spin systems and by comparisons of the angular dependence of the coupling constants [31]. In the ^1^H NMR spectrum of **1**, H-4 showed a triplet of doublets (*J* = 8.4, 3.2 Hz), indicating the *β*-axial position of H-4 to a *β*-equatorially oriented H-3 at *δ*_H_ 5.50 (^1^H, q, *J* = 3.7 Hz) and an *α*-axially oriented H-5 at *δ*_H_ 4.21 (1H, td, *J* = 9.0, 4.0 Hz), which were further proved by the NOESY correlations of H-3/H-4 (Figure 4) [31,32]. In general, the ^1^H NMR protons of H-2_ax_ and H-6_ax_ appeared as dd signals for the spin–spin coupling constants derived from *J*_2ax,2eq_, *J*_2ax,3_ and *J*_6ax,6eq_, *J*_6ax,5_, respectively, while a pattern of ddd signals existed for H-2_eq_ and H-6_eq_, attributed to the extra ^4^*J*_2eq,6eq_ *W* coupling (nearly 2.0 Hz) [31,32], which was always overlapped in this work. Combined with the HSQC correlation, H-6_ax_ at *δ*_H_ 1.97 (1H, dd, *J =* 13.5, 9.6 Hz) and H-6_eq_ at *δ*_H_ 2.17 (^1^H, dd, *J* = 13.5, 3.6 Hz) could be well distinguished by the great difference in the coupling constants between *J*_6ax,5_ (axial–axial coupling constant, 9–11 Hz) and *J*_6eq,5_ (axial–equatorial coupling constant, 3–5 Hz) [31]. Meanwhile, the close coupling constant between *J*_2ax,3_ (axial–equatorial coupling constant, 3–5 Hz) and *J*_2eq,3_ (equatorial–equatorial coupling constant, 4–5 Hz), H-2_ax_ at *δ*_H_ 2.24 (1H, dd, *J* = 14.7, 3.6 Hz) was differentiated by the NOESY correlation of H-2_ax_/H-6_ax_, and the other proton associated to H-2_ax_ in the HSQC spectrum at *δ*_H_ 2.18 (1H, overlapped) was assigned to H-2_eq_. With the aid of 2D NMR data, all of the ^1^H and ^13^C NMR signals of **1** were assigned as shown in Table 2.

In the HMBC spectrum, no correlation was observed between the oxygenated protons in the quinic acid moiety and the carbonyl carbon at *δ*_C_ 167.9, so the vanilloyl group attached to C-3 was deduced by the downfield shift of H-3 (*δ*_H_ 5.50) according to the substituent chemical shifts effect [31], together with the NOESY correlations of H-5_ax_/H-2′ and H-6′. Meanwhile, the chair conformation of the quinic acid unit was supported by the large axial–axial coupling constants of *J*_4,5_ and *J*_5,6ax_, and these key NOESY correlations are shown in Figure 4. In conclusion, the structure of compound **1** was deduced as 3-*O*-vanilloylquinic acid.

The NMR data of compound **2** was similar to those of **1**, except for the appearance of an extra methoxy group in **2**, which was further supported by the molecular formula C_16_H_20_O_10_ deduced by the HRESIMS ion peak [M–H]^–^ at *m/z* 371.0984 (calculated for 371.0978). In the ^1^H NMR and ^13^C NMR data (Table 2) of **2**, except for the typical signals belong to the quinic acid unit similar to compound **1**, two symmetric aromatic protons at *δ*_H_ 7.43 (2H, s, H-2′/H-6′) and two methoxy groups at *δ*_H_ 3.90 (6H, s, 3′/5′-OCH_3_) were observed. The HMBC cross-peaks (Figure 3) from OCH_3_ to C-3′/5′ and from H-2′/H-6′ to C-1′, C-3′/5′, C-4′, and C-7′ disclosed the presence of a syringoyl moiety in **2** [28]. An assignment of H-4 was ascertained at *δ*_H_ 3.69 (1H, dd, *J* = 8.8, 3.3 Hz) by the ^1^H−^1^H COSY correlations of H-3/H-4/H-5, H-3 at *δ*_H_ 5.51 (1H, q, *J* = 3.6 Hz), and H-5 at *δ*_H_ 4.23 (1H, td, *J* = 9.6, 4.2 Hz), which were assigned by the same coupling constant pattern as **1**. Similarly, H-6_ax_ at *δ*_H_ 1.96 (1H, dd, *J* = 13.6, 10.1 Hz) was determined by its larger coupling constant, and *δ*_H_ 2.25 was assigned to H-2_ax_ by the NOESY correlation of H-2_ax_/H-6_ax_ (Figure 4). Meanwhile, H-2_eq_ and H-6_eq_ were well defined by the associated signals of H-2_ax_ and H-6_ax_ in the HSQC spectrum, respectively.

In the HMBC spectrum, H-3 (*δ*_H_ 5.51) was correlated with C-7′ (*δ*_C_ 167.9), which unambiguously indicates that a syringoyl group was located at C-3, which was further confirmed by the NOESY correlations of H-5_ax_/H-2′, H-6′. Moreover, the key NOESY correlations of **2**, as shown in Figure 4, along with the large vicinal coupling constants of *J*_4,5_ and *J*_5,6ax_, proved the chair conformation of the quinic acid unit as **1**. Therefore, compound **2** was defined as 3-*O*-syringoylquinic acid.

Compound **3** was isolated as a colorless oil, and its positive HRESIMS data indicated an [M–H]^–^ ion peak at *m/z* 551.1401 (calculated for 551.1401) corresponding to the molecular formula C_25_H_28_O_14_. A comparison of the ^1^H NMR and ^13^C NMR data (Table 2) of **3** and **2** revealed that the two compounds possessed quite similar structures, and the main differences lay in the two extra symmetric aromatic protons and two methoxy groups in the ^1^H NMR spectrum of **3**, which implies that compound **3** was substituted by two syringoyl groups, supported by the presence of two sets of carbon signals in the ^13^C NMR spectrum belonging to the syringoyl group. Additionally, the signals of the two syringoyl groups could not be well distinguished for their similar magnitudes. For the quinic acid unit, H-4 (*δ*_H_ 5.30, 1H, dd, *J* = 8.2, 3.5 Hz) was well defined by the ^1^H−^1^H COSY correlations of H-3/H-4/H-5, and H-3 (*δ*_H_ 5.82, 1H, dt, *J* = 6.3, 3.5 Hz) could be determined by its characteristic axial–equatorial and equatorial–equatorial coupling constants with adjacent protons [32]. The remaining oxygenated protons at *δ*_H_ 4.39 (1H, brs) were assigned to H-5, but it is noteworthy that H-5 appeared as a broad singlet with a large half-width value (*J*_hw/2_) of 18.3 Hz, which was nearly identical to the reported value to differentiate H-3 (*J*_hw/2_ 8–10 Hz) and H-5 (*J*_hw/2_ 20–28 Hz) when their signals appear as singlets [32,33]. H_2_-6 and H_2_-2 were distinguished by the ^1^H−^1^H COSY correlations of H-5/H-6 and H-2/H-3, respectively. Due to the overlap of H-2_eq_/H-6_ax_ at *δ*_H_ 2.19, H-2_ax_ (*δ*_H_ 2.43, 1H, dd, *J* = 14.6, 3.6 Hz) was preferentially identified by the NOESY correlations of H-2_ax_/H-4_ax_, and H-6_eq_ (*δ*_H_ 2.25, 1H, dd, *J* = 13.7, 3.7 Hz) was subsequently deduced by its small coupling constants of *J*_6eq,5_ and the NOESY correlation of H-6_eq_/H-5 (Figure 4).

Due to the absence of a long-range correlation between carbonyl carbon at *δ*_C_ 167.1/167.3 and the oxygenated protons of the quinic acid unit in the HMBC spectrum of **3**, the two syringoyl groups linked to C-3 and C-4 were ascertained by the downfield shift of H-3 (*δ*_H_ 5.82) and H-4 (*δ*_H_ 5.30) compared to the corresponding chemical shift data of quinic acid [31]. The assumption of the chair conformation of the quinic acid unit was evidenced by the NOESY correlations shown in Figure 4. Thus, compound **3** was elucidated as 3,4-di-*O*-syringoylquinic acid.

Those nine known compounds isolated from *C. spinarum* were all identified by the NMR data as neochlorogenic acid (**4**) [34], cryptochlorogenic acid (**5**) [35], 3,4-dicaffeoylquinic acid (**6**) [36], 3,5-dicaffeoylquinic acid (**7**) [37], 4,5-dicaffeoylquinic acid (**8**) [31], methyl 3,5-dicaffeoylquinate (**9**) [36], methyl 4,5-dicaffeoylquinate (**10**) [38], 4-*O*-caffeoyl-3-*O*-syringoylquinic acid (**11**) [29], and 4-*O*-caffeoyl-3-*O*-vanilloylquinic acid (**12**) [28], respectively. In addition, all of the above identifications were further confirmed by comparison with the spectroscopic data reported in the literature.

### 3.3. Antioxidant Activity Assays for the Isolated Compounds

In order to further confirm those potential active components screened out with UF–LC–MS targeting SOD, the free radical scavenging capacities of all isolated compounds were evaluated by making use of the stable free radical DPPH, the strong purple color of which discolors when reacted with compounds possessing antioxidant ability. The color change in DPPH can be measured spectrophotometrically at 517 nm as an index of the free radical scavenging capacities of tested compounds [39]. As a result, compounds **4** and **6**–**12** exhibited DPPH radical scavenging capacities of varying degrees, while the other compounds did not show obvious activity. As per the IC_50_ values shown in Table 3, compounds **4** and **6**–**11** possessed stronger DPPH radical scavenging capacities than the positive control, vitamin C, as the IC_50_ values ranked as follows: **9** < **10** < **6** < **8** < **7** < **4** < **11** < vitamin C < **12**.

A ferric reducing antioxidant power (FRAP) assay was performed to evaluate the total antioxidant capacity of these isolated compounds, in which the intense blue color of the ferrous TPTZ complex in the reduction from ferric TPTZ could be detected at 593 nm, and the standard Fe^2+^ equivalent (mmol Fe^2+^/g) was used to express the antioxidant power [23]. Similar to the DPPH results, compounds **4** and **6**–**11** showed significant ferric reducing antioxidant power, among which the total antioxidant capacities of compounds **6**, **8**, and **10** were even stronger than the positive control, vitamin C (Table 3).

Combining the results of UF–LC–MS screening and the antioxidant verification experiments, peaks with higher EF values (peaks 8, 10, 11, and 17 corresponding to compounds **6**, **7**, **9**, and **11**, respectively; except for peak 15) showed better antioxidant activities in both the DPPH and FRAP methods, while those ones with lower EF values (peaks 1 and 2 corresponding to the compounds **1** and **2**, respectively; except for peak 14 not isolated) did not show antioxidant potential in either assay. In other words, the above antioxidant activity validation is roughly consistent with the EF values listed in Table 1, which means that the UF–LC–MS method, to a certain extent, could be an efficient strategy to guide the rapid discovery of bioactive components from complex crude extracts. Certainly, due to the complicated biological factors and the mechanisms of different validation methods, UF screening may show small discrepancies; for example, compound **4** displayed good antioxidant activity but possessed a relatively lower EF value (peak 3, 2.68%), so experimental verification is definitely necessary for further acquisition of a conclusion.

### 3.4. Hepatoprotective Properties of the Selected Compounds

**Figure 5:**
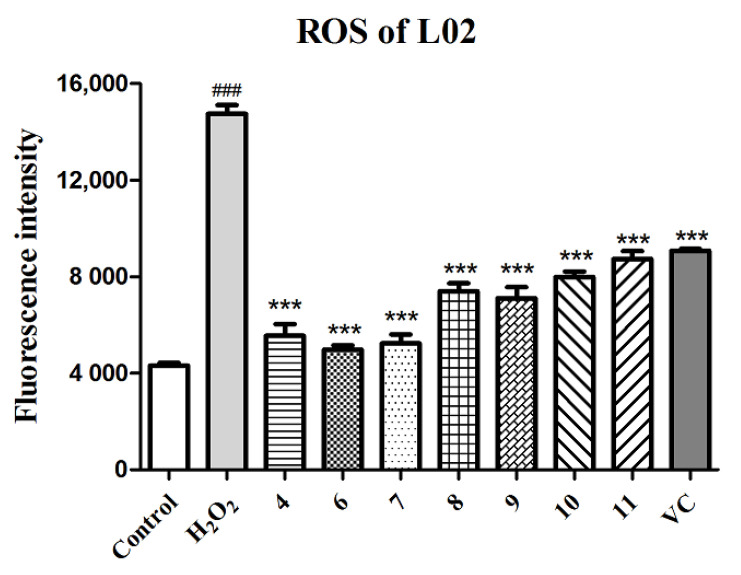
Quantitation of ROS production with compounds **4** and **6**–**11** and the positive control (VC) at 5 µM in the H_2_O_2_-induced L02 cell injury model. The data of the cells treated with compounds only were similar to those of the control group. Data are expressed as means ± SD (*n* = 3). ^###^ *p* < 0.001 compared to the control group and *** *p* < 0.001 compared to the model group.

Many hepatic pathological processes are related to the oxidative stress caused by ROS, such as nonalcoholic fatty liver disease (NAFLD), fibrosis, and hepatocellular carcinoma (HCC) [40]. Thus, compounds **4** and **6**–**11**, screened out and verified as having promising antioxidant activities, were tested for their potential in the protection of hepatic cells from H_2_O_2_-induced oxidative injury. As shown in Table 4, these tested compounds exhibited statistically significant hepatic protective effects of different degrees against H_2_O_2_-induced L02 injury compared to the negative control group, especially at the concentration of 5 μM. Except the improvement in cell viability, the ROS level in the H_2_O_2_-induced L02 cell injury model was also detected by the DCFH-DA fluorescent method with fluorescence image photography and fluorescence intensity measurement. As shown in Figure 5 and Figure S28, L02 cells treated with H_2_O_2_ exhibited an obvious increase in ROS production (*p* < 0.001) compared to the normal control group, while cells pretreated with compounds **4** and **6**–**11** (final concentration of 5 μM) showed a remarkable decrease in ROS levels (*p* < 0.001) in comparison to the model group, which indicated that compounds **4** and **6**–**11** relieved the cell death induced by H_2_O_2_ through eliminating ROS production.

**Figure 6:**
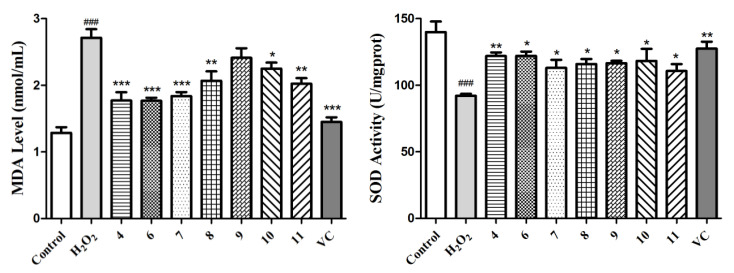
Determination of the MDA levels and SOD activities for compounds **4** and **6**–**11** and the positive control (VC) at 5 µM using the H_2_O_2_-induced L02 cell injury model. The data of the cells treated with compounds only were similar to those of the control group. Data are expressed as means ± SD (*n* = 3). ^###^ *p* < 0.001 compared to the control group and * *p* < 0.05, ** *p* < 0.01, and *** *p* < 0.001 compared to the model group.

Next, we further investigated the MDA level in the culture medium, which was a reflection of the lipid peroxidation levels in the cells and the SOD enzymatic activity of the L02 cells in each group. As exhibited in Figure 6, H_2_O_2_ induced an obvious increase in the MDA level and decrease in the SOD activity (*p* < 0.001), whereas this phenomenon was improved markedly in the compound- or vitamin C-treated groups. Taking all of these data into consideration, compounds **4** and **6**–**11** can protect L02 cells from oxidative death by reducing the ROS and MDA levels and improving the SOD activity. It has also been reported that these acyl quinic acid derivatives are a group of phenolic secondary metabolites, among which chlorogenic acid, as the most well-known one, has been used for its health benefits and has also been found to possess various pharmacological functions, such as anti-oxidative, anti-inflammatory, and anti-hypertension activities [41]. In this sense, our work further affirms its health benefits, and expands the application of acyl quinic acid derivatives as a new type of natural compound for potential hepatic protection.

## 4. Conclusions

In order to explore the responsible chemical components in the root barks of *C. spinarum* and their action mechanisms for antioxidative and hepatoprotective activities, SOD enzyme-based UF–LC/MS screening was firstly used to reveal that acyl quinic acid derivatives possess potential bio-affinity to SOD, and the following phytochemical investigation led to the isolation of 12 acyl quinic acid derivatives (**1**–**12**), including three new ones (**1**–**3**). Then, subsequent in vitro anti-oxidative assays further revealed that compounds **4** and **6**–**11** possess promising antioxidant activities with lower IC_50_ values than vitamin C. In consideration of the key role oxidative stress plays in different hepatic pathological processes, the H_2_O_2_-induced L02 cell oxidative injury model was further applied to evaluate the hepato-protective properties of these selected compounds based on the antioxidative activities. It turned out that compounds **4** and **6**–**11** could attenuate H_2_O_2_-induced cell death to different degrees by reducing ROS and MDA production and increasing the SOD activity in L02 cells. In conclusion, this study not only provides some new evidence to support the traditional use of *C. spinarum* for antioxidative and hepatic protection, but also offers potential antioxidative and hepatoprotective candidate compounds from *C. spinarum* for further application. To the best of our knowledge, this is the first report using an efficient ultrafiltration screening approach with SOD as the target to simultaneously screen and identify the antioxidant ligands directly from a complex mixture of C*. spinarum*, and revealing its corresponding active compounds with good antioxidative and hepatoprotective activities.

## Figures and Tables

**Figure 2:**
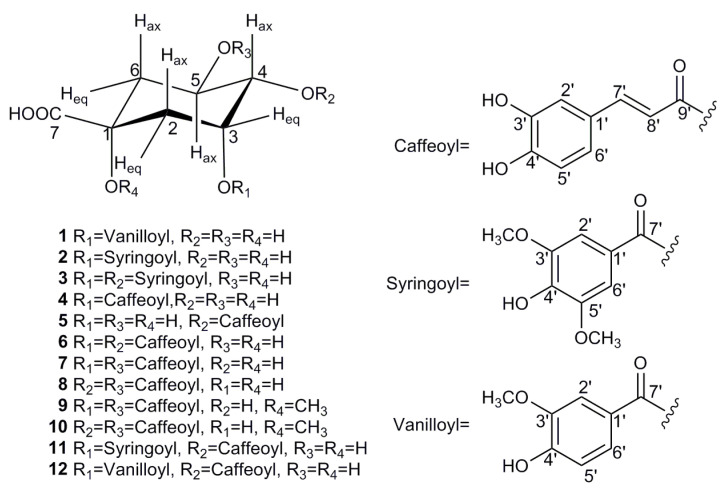
Chemical structures of compounds **1**–**12** isolated from *C. spinarum*.

**Table 1:** Identification of potential SOD ligands in the EA fraction of *C. spinarum*.

Peak	Rt * (min)	EF * (%)	[M–H]^–^ (*m/z*)	Characteristic Fragments (*m/z*)	Identification (Corresponding Isolated Compound)
**P1**	10.24	−0.24	341	191, 167	3-*O*-vanilloylquinic acid (compound **1**)
**P2**	11.13	2.34	371	197, 191	3-*O*-syringoylquinic acid (compound **2**)
**P3**	11.68	2.68	353	191, 179, 135	neochlorogenic acid (compound **4**)
**P4**	14.46	2.96	353	191, 179, 135	chlorogenic acid (NI *)
**P5**	15.34	2.67	353	191, 179, 135	cryptochlorogenic acid (compound **5**)
**P6**	17.27	3.00	551	197, 191	3,4-di-*O*-syringoylquinic acid (compound **3**)
**P7**	23.67	3.11	551	197, 191	di-*O*-syringoylquinic acid isomer (NI)
**P8**	24.18	3.16	533	353, 335, 197, 191, 179	4-*O*-caffeoyl-3-*O*-syringoylquinic acid (compound **11**)
**P9**	24.50	2.65	503	341, 191, 179	4-*O*-caffeoyl-3-*O*-vanilloylquinic acid (compound **12**)
**P10**	24.75	3.16	515	353, 335, 191, 179	3,4-dicaffeoylquinic acid (compound **6**)
**P11**	25.47	3.30	515	353, 335, 191, 179	3,5-dicaffeoylquinic acid (compound **7**)
**P12**	26.06	3.00	551	197, 191	di-*O*-syringoylquinic acid isomer (NI)
**P13**	26.84	3.06	515	353, 335, 191, 179	4,5-dicaffeoylquinic acid (compound **8**)
**P14**	27.31	2.48	503	353, 341, 191, 179	caffeoyl-vanilloylquinic acid isomer (NI)
**P15**	27.70	3.51	559	335, 223, 197, 191, 179	caffeoyl-sinapoylquinic acid isomer (NI)
**P16**	28.88	3.07	529	367, 179, 161	methyl 4,5-dicaffeoylquinate (compound **10**)
**P17**	31.17	3.53	529	367, 179, 161	methyl 3,5-dicaffeoylquinate (compound **9**)

* Rt, retention time; EF, enrichment factor; NI, non-isolated compound.

**Table 2:** ^1^H NMR and ^1^^3^C NMR spectroscopic data of the new compounds **1**–**3** *^a^.*

	1		2		3	
Position	*δ*_H_, (*J* in Hz)	*δ*_C_, Type	*δ*_H_, (*J* in Hz)	*δ*_C_, Type	*δ*_H_, (*J* in Hz)	*δ*_C_, Type
1		75.6, C		75.6, C		75.4, C
2ax	2.24, dd (14.7, 3.6)	37.0, CH_2_	2.25, dd (14.9, 3.6)	37.0, CH_2_	2.43, dd (14.6, 3.6)	37.9, CH_2_
2eq	2.18, overlap		2.18, m (overlap)		2.19, m, overlap	
3	5.50, q (3.7)	73.2, CH	5.51, q (3.6)	73.4, CH	5.82, dt (6.3, 3.5)	70.5, CH
4	3.70, dd (8.7, 3.2)	75.0, CH	3.69, dd (8.8, 3.3)	75.2, CH	5.30, dd (8.2, 3.5)	76.1, CH
5	4.21, td (9.0, 4.0)	68.5, CH	4.23, td (9.6, 4.2)	68.3, CH	4.39, brs	67.0, CH
6eq	2.17, dd (13.5, 3.6)	41.5, CH_2_	2.18, m (overlap)	41.8, CH_2_	2.25, dd (13.7, 3.7)	41.2, CH_2_
6ax	1.97, dd (13.5, 9.6)		1.96, dd (13.6, 10.1)		2.19, m, overlap	
7		179.0, C		178.6, C		179.5, C
1’/1” *^b^*		123.3, C		122.2, C		121.4/121.5, C
2’/2” *^b^*	7.67, d (1.9)	114.1, CH	7.43, s	108.5, CH	7.28/7.33, s	108.3/108.3, CH
3’/3” *^b^*		148.6, C		148.7, C		148.8/148.9, C
4’/4” *^b^*		152.6, C		141.6, C		141.9/142.0, C
5’/5” *^b^*	6.84, d (8.2)	115.7, CH		148.7, CH		148.8/148.9, C
6’/6” *^b^*	7.63, dd (8.2, 1.9)	125.4, CH	7.43, s	108.5, CH	7.28/7.33, s	108.3/108.3, CH
7’/7” *^b^*		167.9, C		167.9, C		167.1/167.3, C
8’/8”						
9’/9”						
3’-OCH_3_	3.91, s	56.4, CH_3_	3.90, s	56.8, CH_3_	3.76/3.80, s	56.5/56.7, CH_3_
5’-OCH_3_			3.90, s	56.8, CH_3_	3.76/3.80, s	56.5/56.7, CH_3_
3”- OCH_3_				56.8, CH_3_	3.76/3.80, s	56.5/56.7, CH_3_
5”- OCH_3_				56.8, CH_3_	3.76/3.80, s	56.5/56.7, CH_3_

*^a^* Data (*δ*) were measured in CD_3_OD by 600 MHz for ^1^H NMR and 150 MHz for ^13^C NMR. The assignments were based on DEPT, ^1^H−^1^H COSY, HSQC, and HMBC experiments. *^b^* Data for H-1’−H-7’ and C-1’–C-7’ of compounds **1** and **2**.

**Table 3:** The antioxidant activities of compounds **1**–**12** and vitamin C with the DPPH and FRAP assays.

Compound No.	DPPH	FRAP
	IC_50_ ± SD (µM)	mmol Fe^2+^/g
**1**	>100	0.4 ± 0.0
**2**	>100	0.8 ± 0.0
**3**	>100	1.0 ± 0.0
**4**	16.5 ± 2.2	6.3 ± 0.2
**5**	>100	1.3 ± 0.0
**6**	11.2 ± 0.9	10.3 ± 0.2
**7**	16.4 ± 0.5	4.8 ± 0.2
**8**	11.3 ± 0.1	12.7 ± 1.2
**9**	4.0 ± 0.3	7.8 ± 0.0
**10**	4.9 ± 0.3	11.4 ± 0.6
**11**	25.3 ± 0.6	3.9 ± 0.1
**12**	45.2 ± 1.5	2.7 ± 0.1
Vitamin C	25.6 ± 0.3	8.1 ± 0.1

**Table 4:** Hepatoprotective effects of compounds **4** and **6**–**11** against H_2_O_2_-induced oxidative injury in L02 cells. ^a^

	Test Concentration (μM)			300 μM
Compound	25	5	1	H_2_O_2_
**4**	80.4 ± 2.6 *^d^*	86.1 ± 0.5 *^d^*	80.7 ± 2.0 *^c^*	70.22 ± 0.3
**6**	58.8 ± 0.9	82.0 ± 0.9 *^d^*	83.8 ± 1.4 *^d^*	70.22 ± 0.3
**7**	75.5 ± 0.9 *^b^*	81.4 ± 1.4 *^c^*	77.7 ± 0.4 *^b^*	70.22 ± 0.3
**8**	63.7 ± 2.3	78.4 ± 0.7 *^b^*	77.6 ± 1.4 *^b^*	70.22 ± 0.3
**9**	77.7 ± 5.6	79.1 ± 1.3 *^b^*	74.7 ± 1.7	70.22 ± 0.3
**10**	68.4 ± 2.8	76.8 ± 0.6 *^b^*	77.8 ± 1.4 *^b^*	70.22 ± 0.3
**11**	76.4 ± 1.3 *^b^*	75.3 ± 2.8 *^b^*	79.1 ± 1.3 *^c^*	68.22 ± 1.1
Vitamin C	82.6 ± 2.1 *^d^*	81.7 ± 2.9 *^d^*	81.8 ± 0.9 *^d^*	65.15 ± 0.7

*^a^* The data (cell viability, measured by SRB assay) were normalized and are expressed as a percentage of the control group, which was set to 100%. The data of the cells treated with compounds only were similar to those of the control group. Data ae expressed as means ± SD. Three independent experiments were carried out. *^b^ p* < 0.05,*^c^ p* < 0.01, and *^d^ p* < 0.001 compared to the H_2_O_2_ model group. Vitamin C was used as the positive control.

## Data Availability

All data in this study are included in this article and Supplementary Materials.

## Supplementary Materials

The following are available online at www.mdpi.com/xxx.s1, Figure S1: (+)-HR-ESI–MS spectrum of **1**; Figure S2: ^1^H NMR (600 MHz, CD_3_OD) spectrum of **1**; Figure S3: ^13^C NMR (150 MHz, CD_3_OD) spectrum of **1**; Figure S4: DEPT 135 (150 MHz, CD_3_OD) spectrum of **1**; Figure S5: HSQC spectrum of **1** in CD_3_OD; Figure S6: ^1^H–^1^H COSY spectrum of **1** in CD_3_OD; Figure S7: HMBC spectrum of **1** in CD_3_OD; Figure S8: NOESY spectrum of **1** in CD_3_OD; Figure S9: UV spectrum of **1** in CD_3_OD; Figure S10. (+)-HR-ESI–MS spectrum of 2; Figure S11. ^1^H NMR (600 MHz, CD_3_OD) spectrum of 2; Figure S12: ^13^C NMR (150 MHz, CD_3_OD) spectrum of **2**; Figure S13: DEPT 135 (150 MHz, CD_3_OD) spectrum of **2**; Figure S14: HSQC spectrum of **2** in CD_3_OD; Figure S15: ^1^H–^1^H COSY spectrum of **2** in CD_3_OD; Figure S16: HMBC spectrum of **2** in CD_3_OD; Figure S17: NOESY spectrum of **2** in CD_3_OD; Figure S18: UV spectrum of **2** in CD_3_OD; Figure S19: (+)-HR-ESI–MS spectrum of **3**; Figure S20: ^1^H NMR (600 MHz, CD_3_OD) spectrum of **3**; Figure S21: ^13^C NMR (150 MHz, CD_3_OD) spectrum of **3**; Figure S22: DEPT 135 (150 MHz, CD_3_OD) spectrum of **3**; Figure S23: HSQC spectrum of **3** in CD_3_OD; Figure S24: ^1^H–^1^H COSY spectrum of **3** in CD_3_OD; Figure S25: HMBC spectrum of **3** in CD_3_OD; Figure S26: NOESY spectrum of **3** in CD_3_OD; Figure S27: UV spectrum of **3** in CD_3_OD; Figure S28: ROS production of compounds **4** and **6**–**11** and the positive control (vitamin C) at 5 µM in the H_2_O_2_-induced L02 cell injury model shown in fluorescence images photographed by a cell imaging multi-mode reader (Cytation 1, BioTek; scale bar: 500 µm, 4×).
