# Supplementary material for: Acyl Quinic Acid Derivatives Screened Out from Carissa spinarum by SOD-Affinity Ultrafiltration LC–MS and Their Antioxidative and Hepatoprotective Activities
Source: Antioxidants (Basel). 2021 Aug 18;10(8):1302. doi: 10.3390/antiox10081302 (PMC8389231; doi:10.3390/antiox10081302)
Supplement: Supplementary file 1 [file antioxidants-10-01302-s001.zip › antioxidants-1303631-supplementary.pdf]

Supporting Information for

Acyl quinic acids derivatives screened out from *Carissa spinarum* by SOD-affinity ultrafiltration LC-MS and their antioxidative and hepatoprotective activities

Ye Liu,[1,2,3] Felix Wambua Muema,[1,2,3,4] Yongli Zhang,[1,2,3] and Mingquan Guo[1,2,3,*]

[1] CAS Key Laboratory of Plant Germplasm Enhancement and Specialty Agriculture, Wuhan Botanical Garden, Chinese Academy of Sciences, Wuhan 430074, China

[2] Sino-Africa Joint Research Center, Chinese Academy of Sciences, Wuhan 430074, China

[3] Innovation Academy for Drug Discovery and Development, Chinese Academy of Sciences, Shanghai 201203, China

[4] Graduate University of Chinese Academy of Sciences, Beijing 100049, China

*Corresponding author: CAS Key Laboratory of Plant Germplasm Enhancement and Specialty Agriculture, Wuhan Botanical Garden, Chinese Academy of Sciences, Wuhan 430074, China

orcid.org/0000-0002-6627-5627; Phone: +86-027-87700850;

E-mail: guomq@wbgcas.cn

Content

Figure S1. (+)-HR-ESI-MS spectrum of **1**

Figure S2. ^{1}H NMR (600 MHz, CD_3OD) spectrum of **1**

Figure S3. ^{13}C NMR (150 MHz, CD_3OD) spectrum of **1**

Figure S4. DEPT 135 (150 MHz, CD_3OD) spectrum of **1**

Figure S5. HSQC spectrum of **1** in CD_3OD

Figure S6. ^{1}H-^{1}H COSY spectrum of **1** in CD_3OD

Figure S7. HMBC spectrum of **1** in CD_3OD

Figure S8. NOESY spectrum of **1** in CD_3OD

Figure S9. UV spectrum of **1** in CD_3OD

Figure S10. (+)-HR-ESI-MS spectrum of **2**

Figure S11. ^{1}H NMR (600 MHz, CD_3OD) spectrum of **2**

Figure S12. ^{13}C NMR (150 MHz, CD_3OD) spectrum of **2**

Figure S13. DEPT 135 (150 MHz, CD_3OD) spectrum of **2**

Figure S14. HSQC spectrum of **2** in CD_3OD

Figure S15. ^{1}H-^{1}H COSY spectrum of **2** in CD_3OD

Figure S16. HMBC spectrum of **2** in CD_3OD

Figure S17. NOESY spectrum of **2** in CD_3OD

Figure S18. UV spectrum of **2** in CD_3OD

Figure S19. (+)-HR-ESI-MS spectrum of **3**

Figure S20. ^{1}H NMR (600 MHz, CD_3OD) spectrum of **3**

Figure S21. ^{13}C NMR (150 MHz, CD_3OD) spectrum of **3**

Figure S22. DEPT 135 (150 MHz, CD_3OD) spectrum of **3**

Figure S23. HSQC spectrum of **3** in CD_3OD

Figure S24. ^{1}H-^{1}H COSY spectrum of **3** in CD_3OD

Figure S25. HMBC spectrum of **3** in CD_3OD

Figure S26. NOESY spectrum of **3** in CD_3OD

Figure S27. UV spectrum of **3** in CD_3OD

Figure S28. ROS production of compounds **4**, **6−11** and positive control vitamin C at 5 μM in H_2O_2-induced L02 cell injury model shown in fluorescence images photographed by cell imaging multi-mode reader (Cytation 1, BioTek; scale bar: 500 μm, 4 ×)

Figure S1. (+)-HR-ESI-MS spectrum of **1**

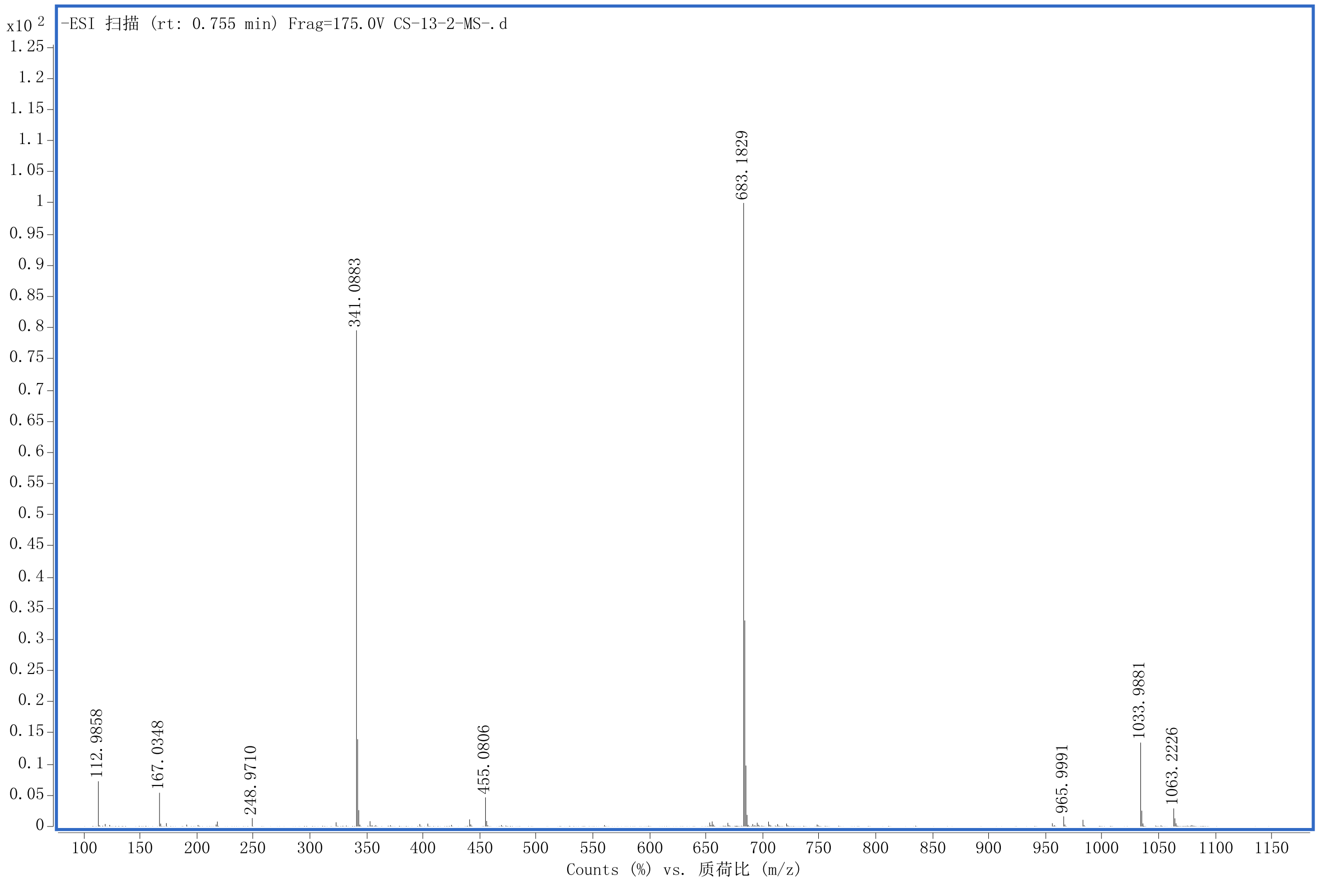

Figure S2. ^{1}H NMR (600 MHz, CD_3OD) spectrum of **1**

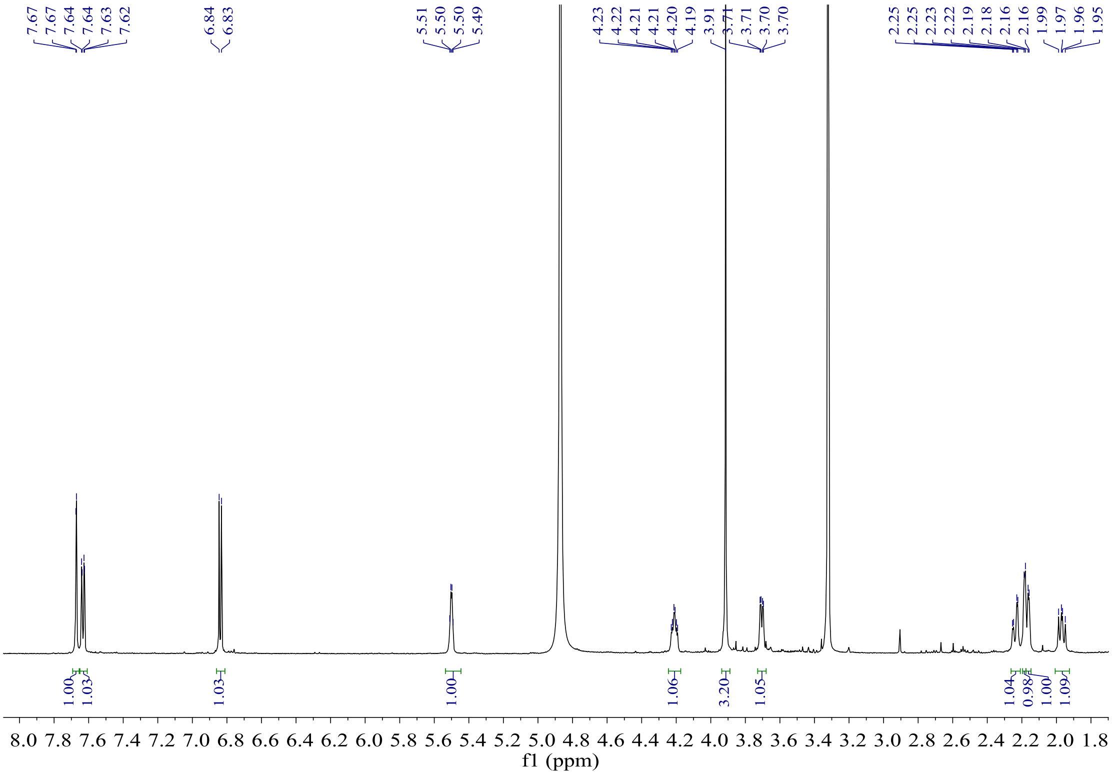

Figure S3. ^{13}C NMR (150 MHz, CD_3OD) spectrum of **1**

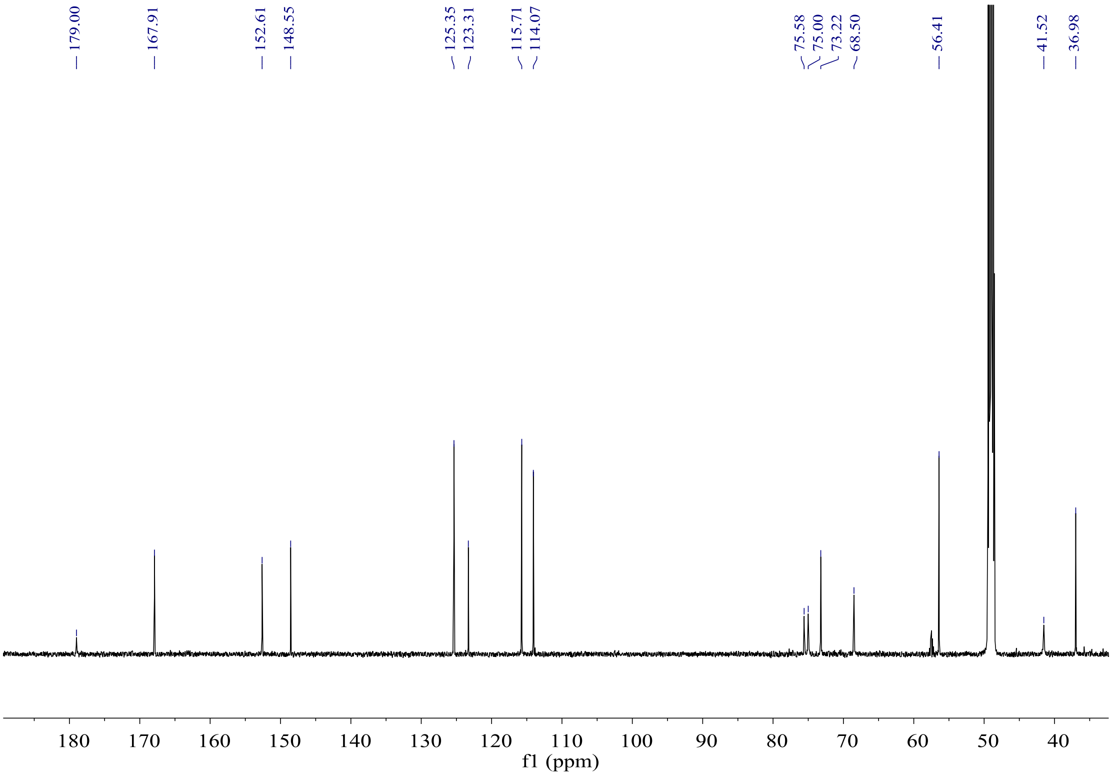

Figure S4. DEPT 135 (150 MHz, CD_3OD) spectrum of **1**

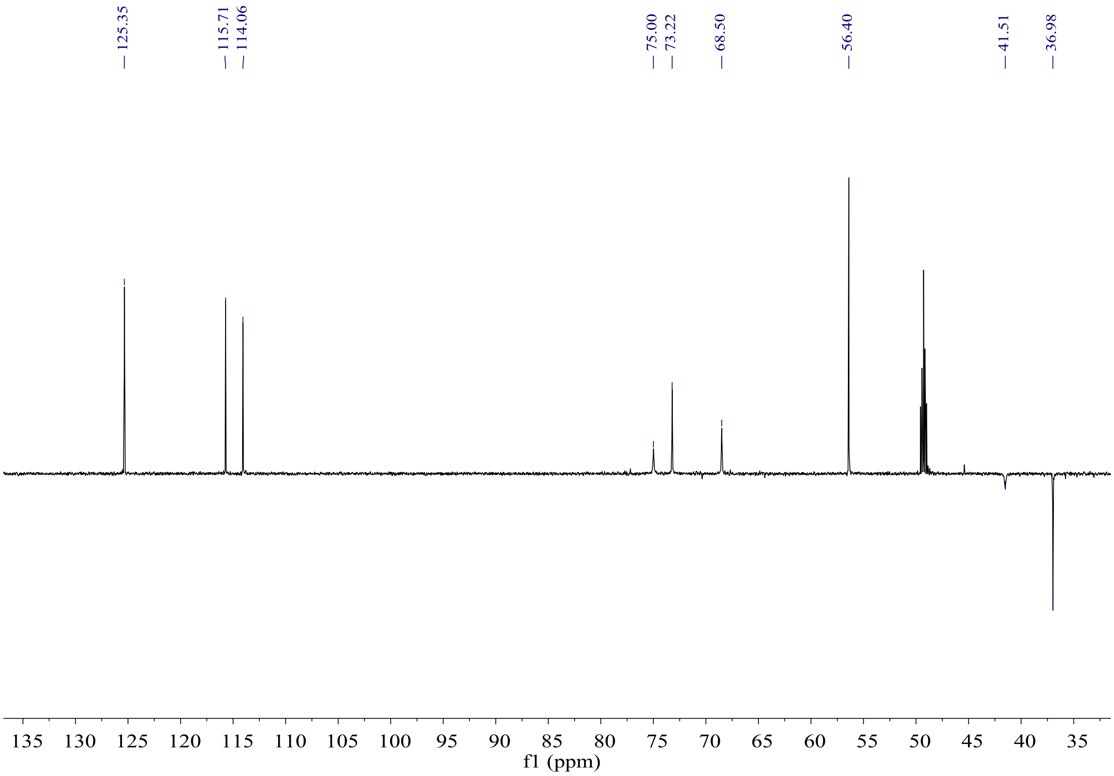

Figure S5. HSQC spectrum of **1** in CD_3OD

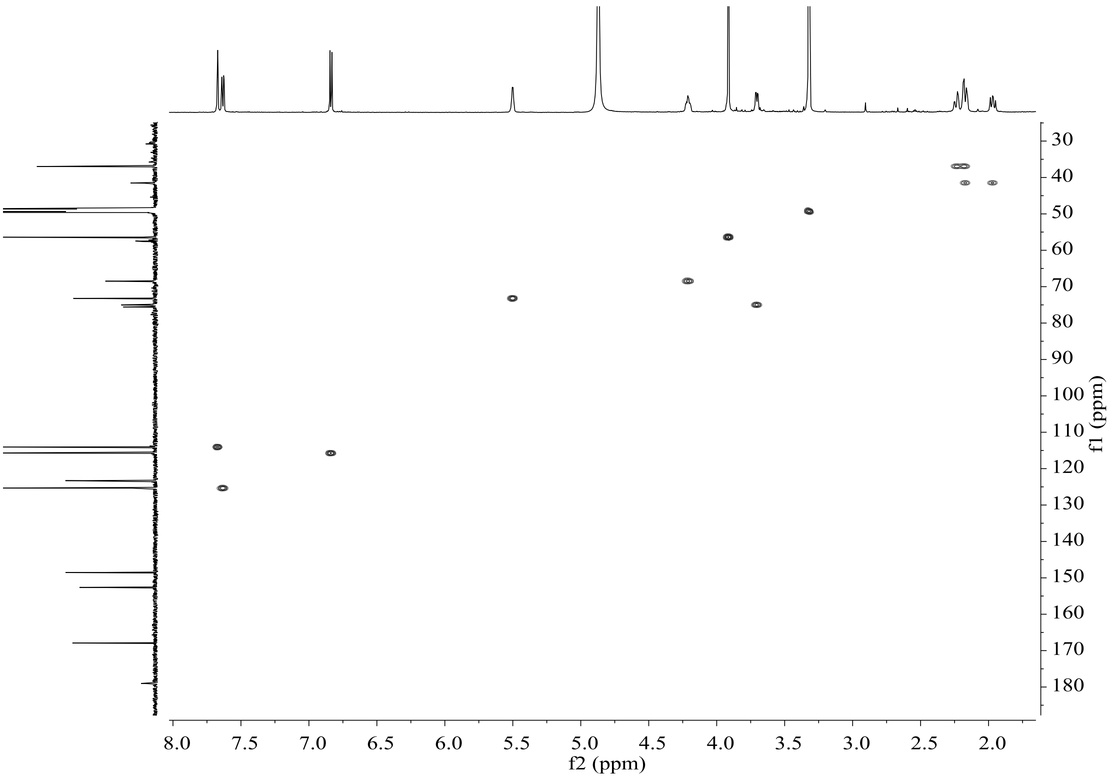

Figure S6. ^1H-^1H COSY spectrum of **1** in CD_3OD

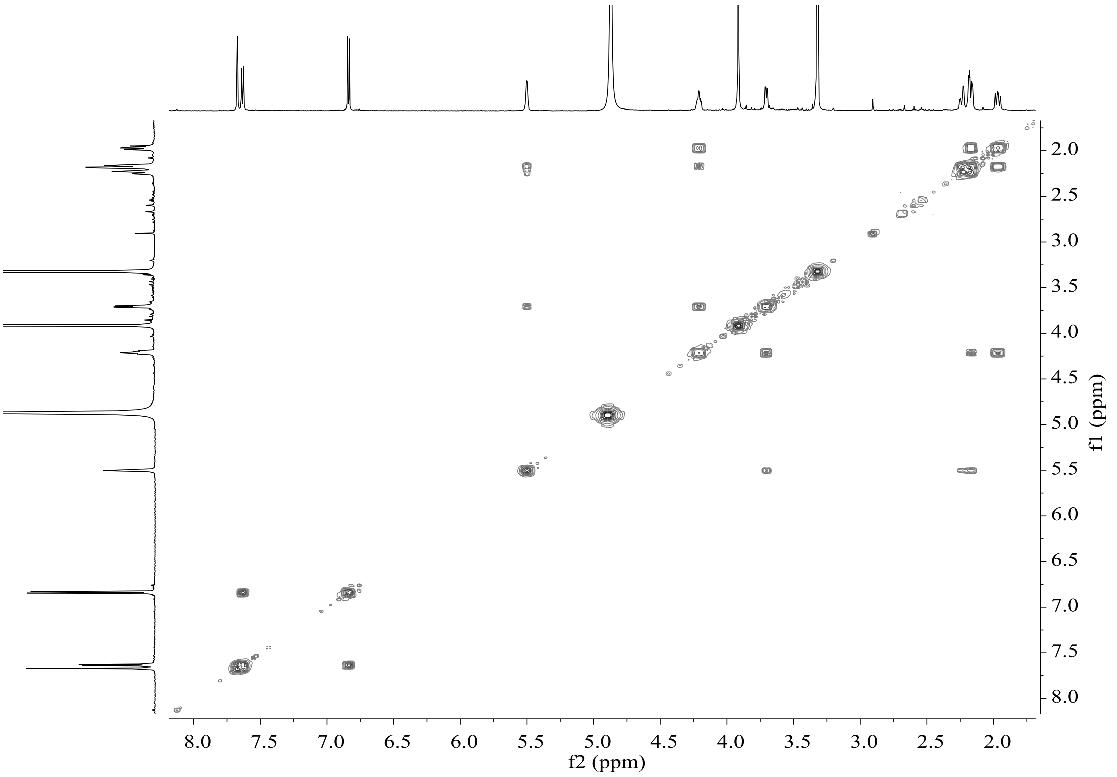

Figure S7. HMBC spectrum of **1** in CD_3OD

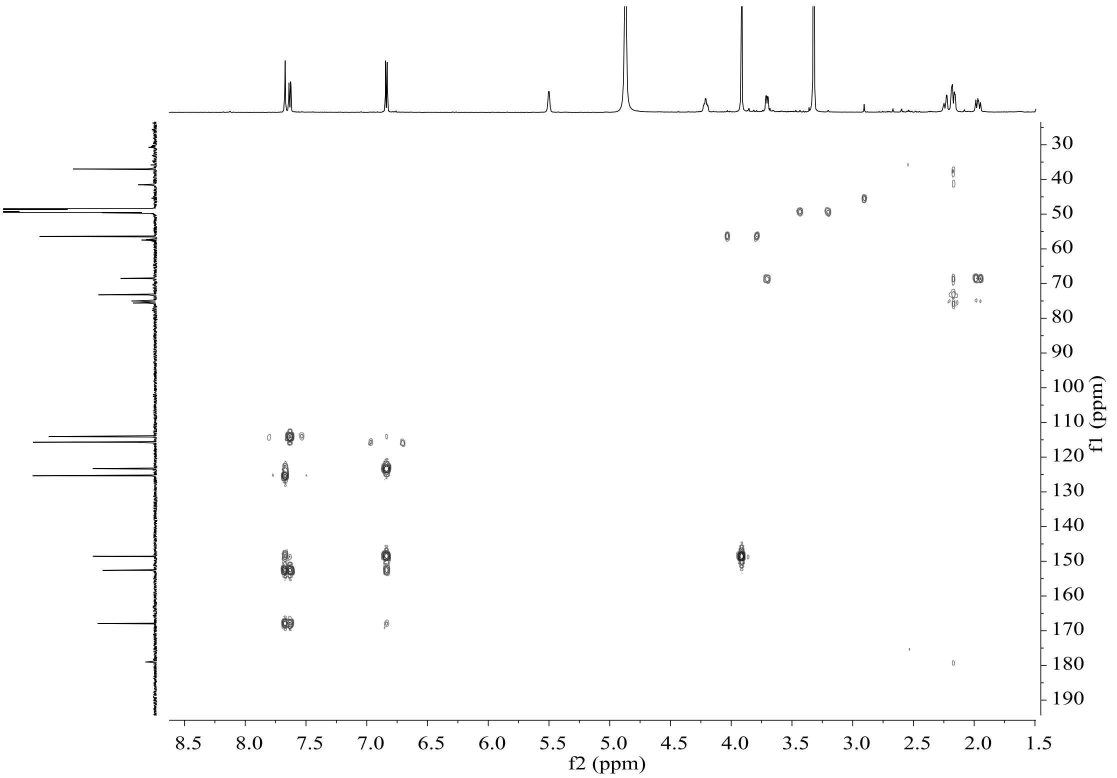

Figure S8. NOESY spectrum of **1** in CD_3OD

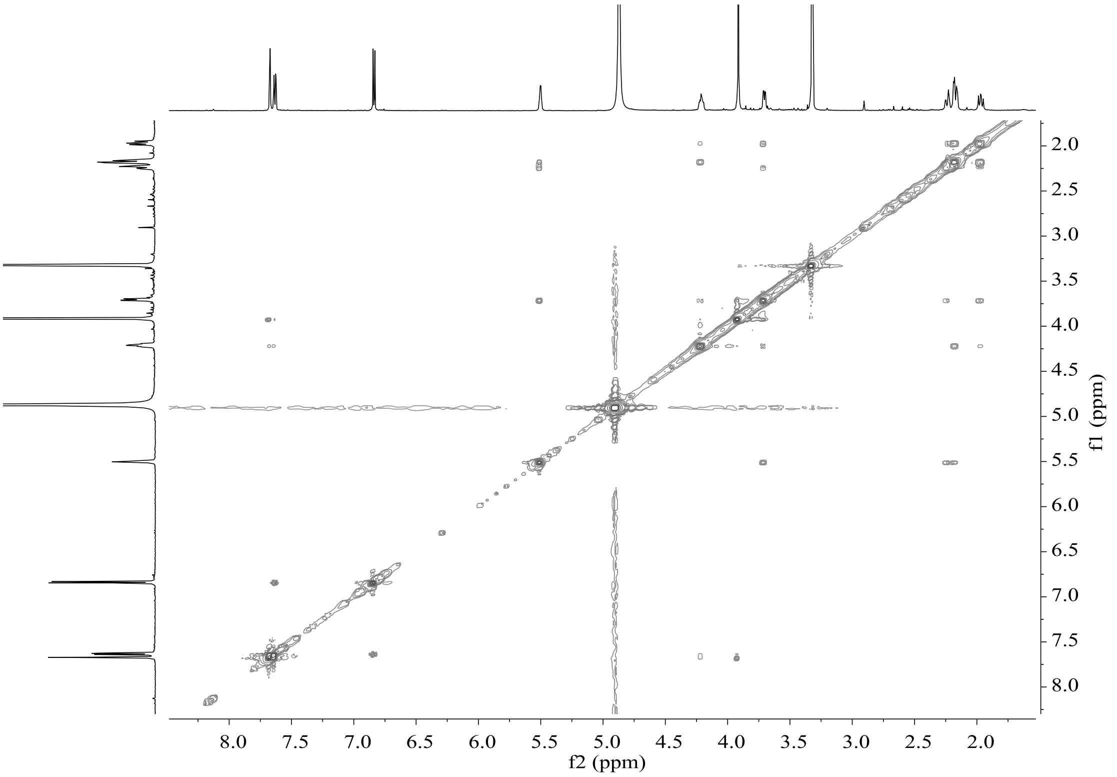

Figure S9. UV spectrum of **1** in CD_3OD

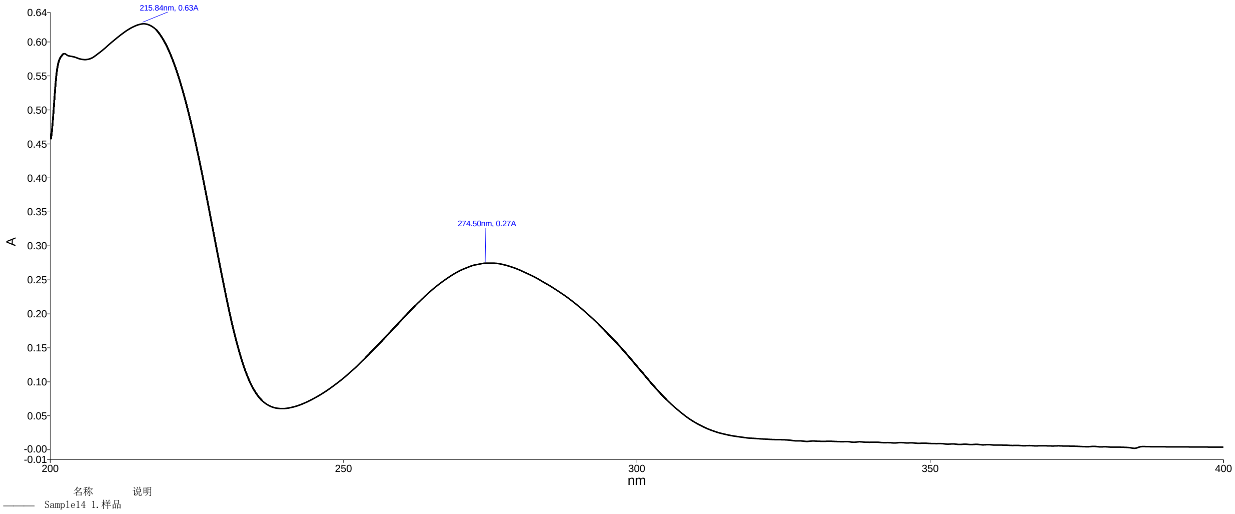

Figure S10. (+)-HR-ESI-MS spectrum of **2**

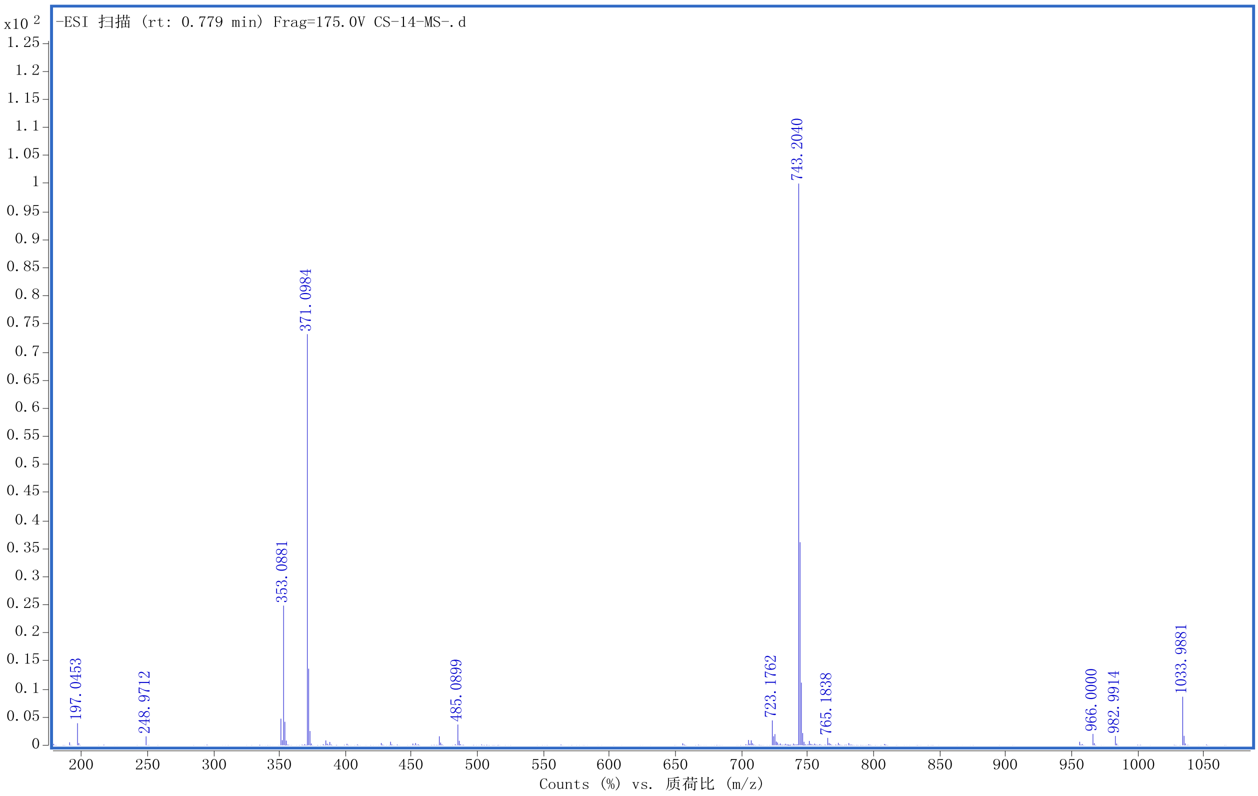

Figure S11. ^{1}H NMR (600 MHz, CD_3OD) spectrum of **2**

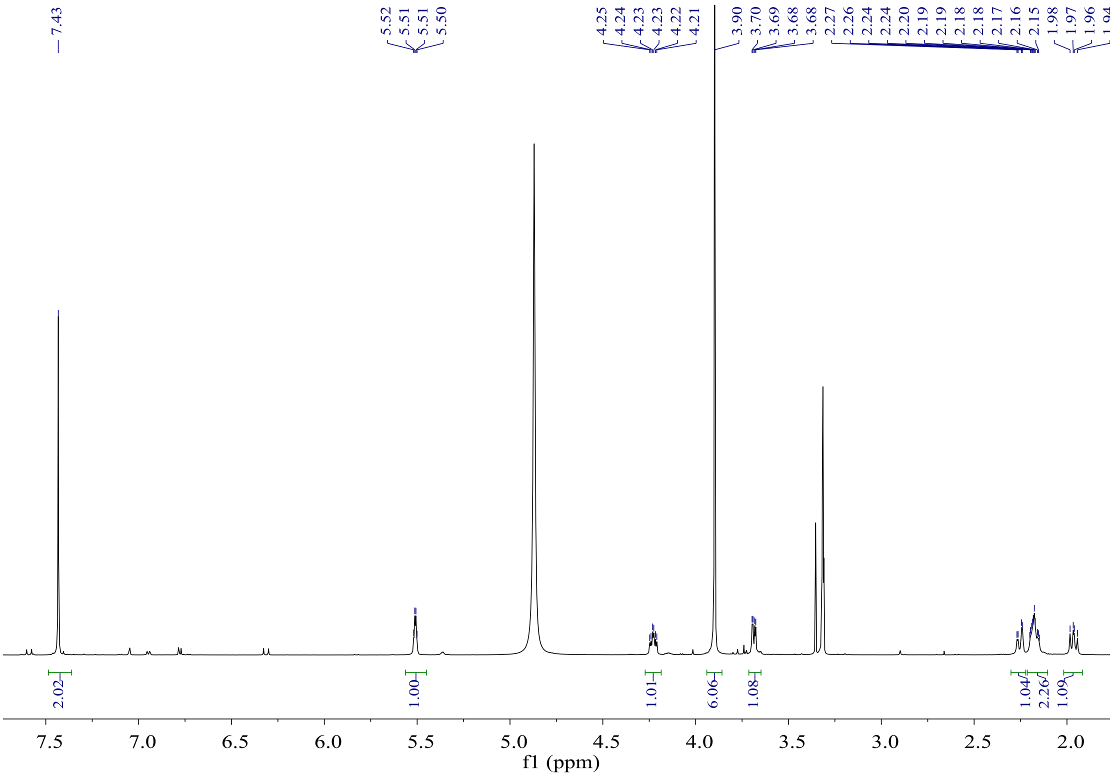

Figure S12. ^{13}C NMR (150 MHz, CD_3OD) spectrum of **2**

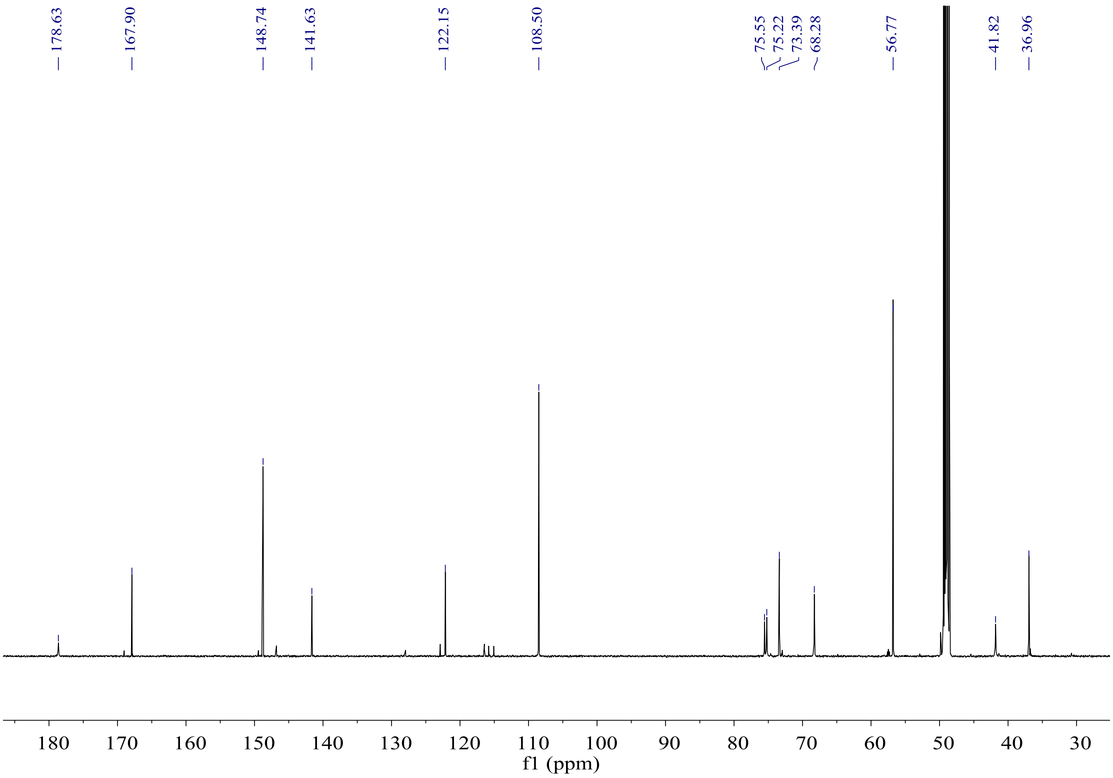

Figure S13. DEPT 135 (150 MHz, CD_3OD) spectrum of **2**

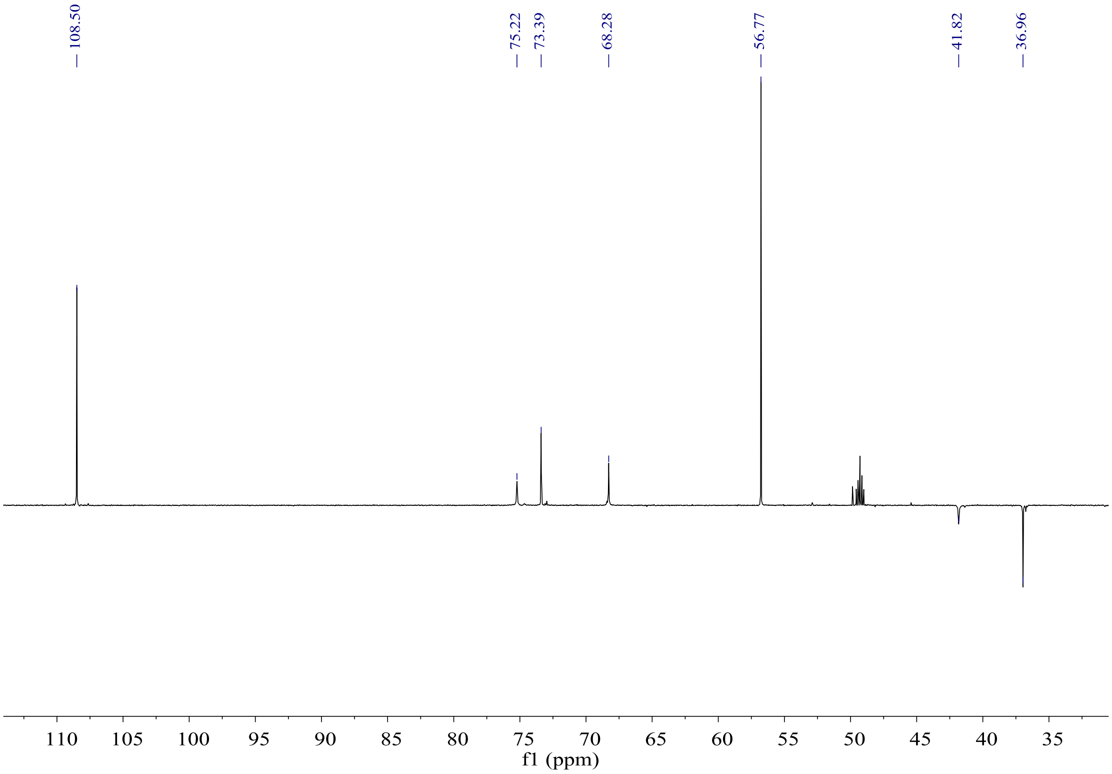

Figure S14. HSQC spectrum of **2** in CD_3OD

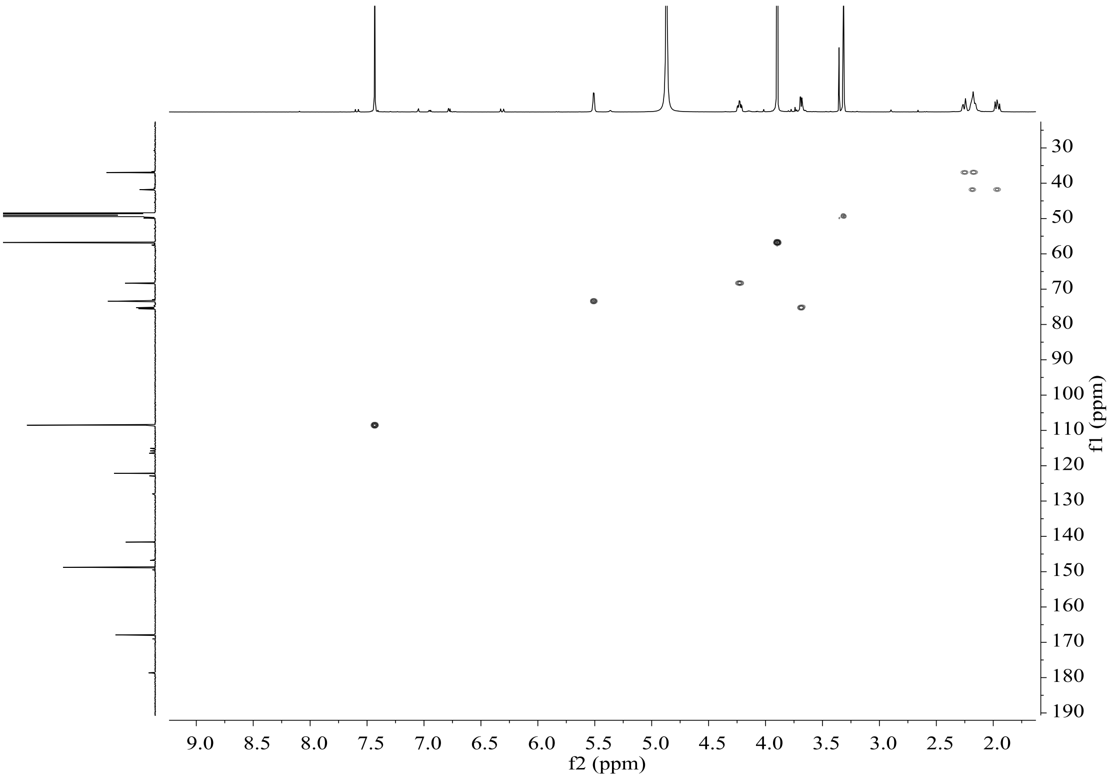

Figure S15. ^{1}H-^{1}H COSY spectrum of **2** in CD_3OD

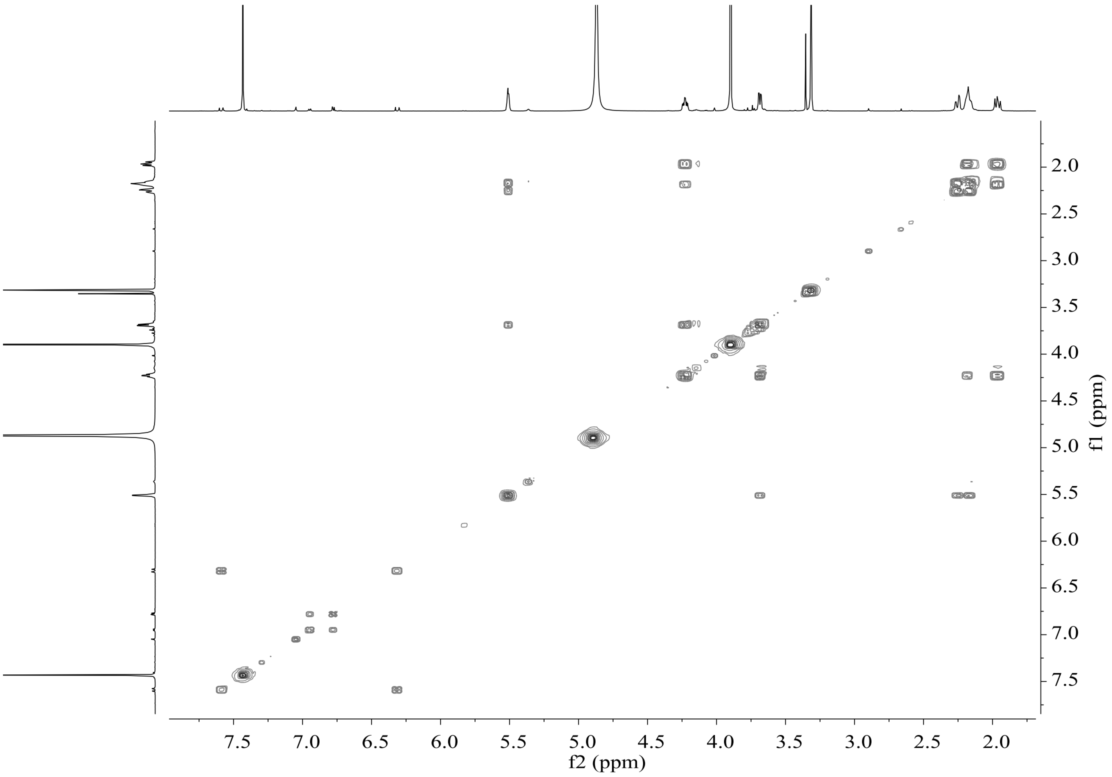

Figure S16. HMBC spectrum of **2** in CD_3OD

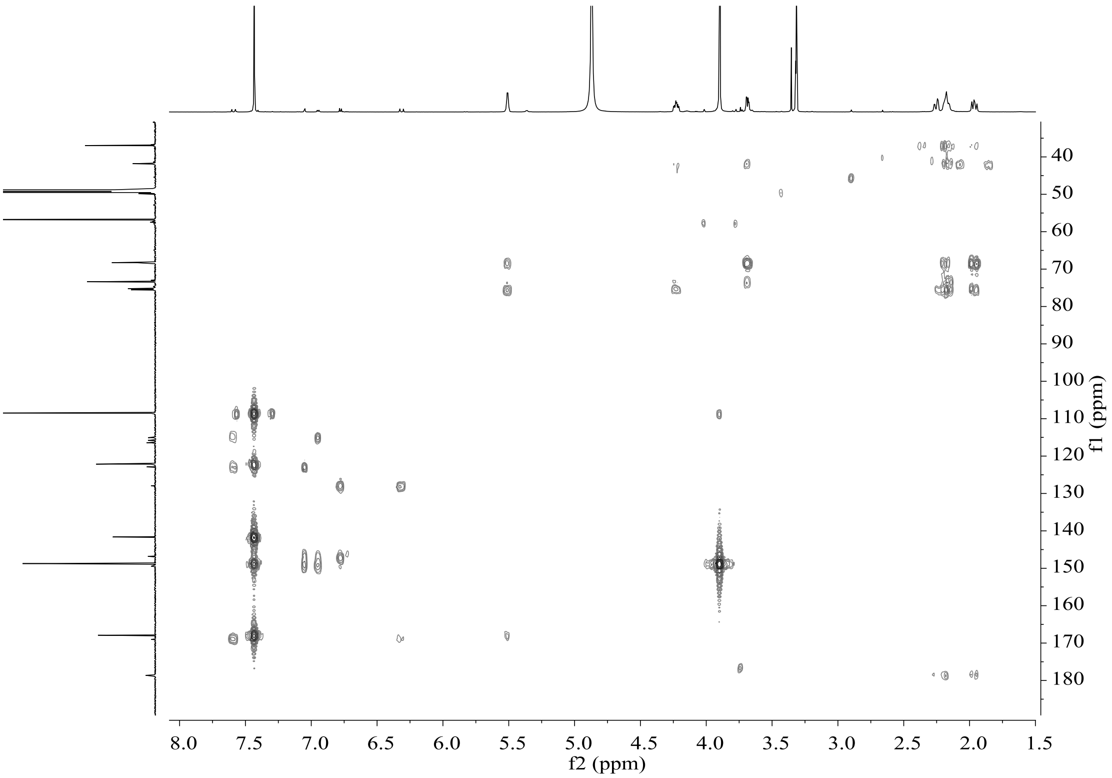

Figure S17. NOESY spectrum of **2** in CD_3OD

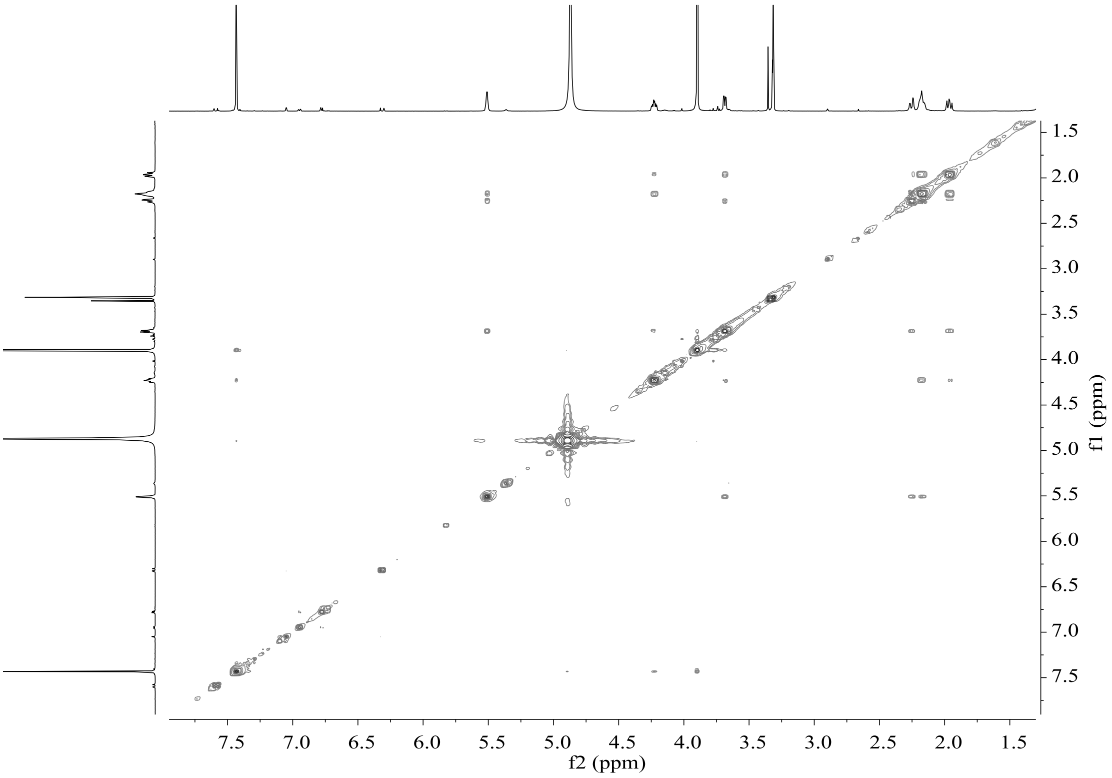

Figure S18. UV spectrum of **2** in CD_3OD

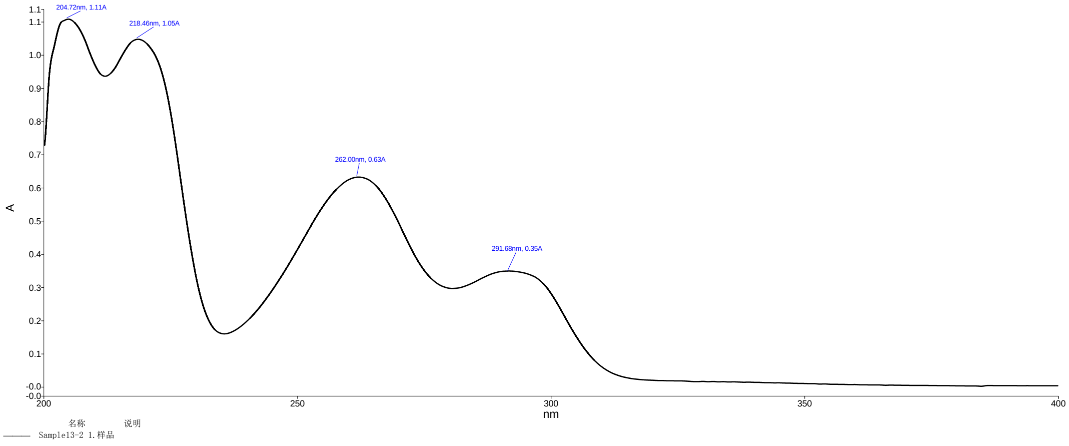

Figure S19. (+)-HR-ESI-MS spectrum of **3**

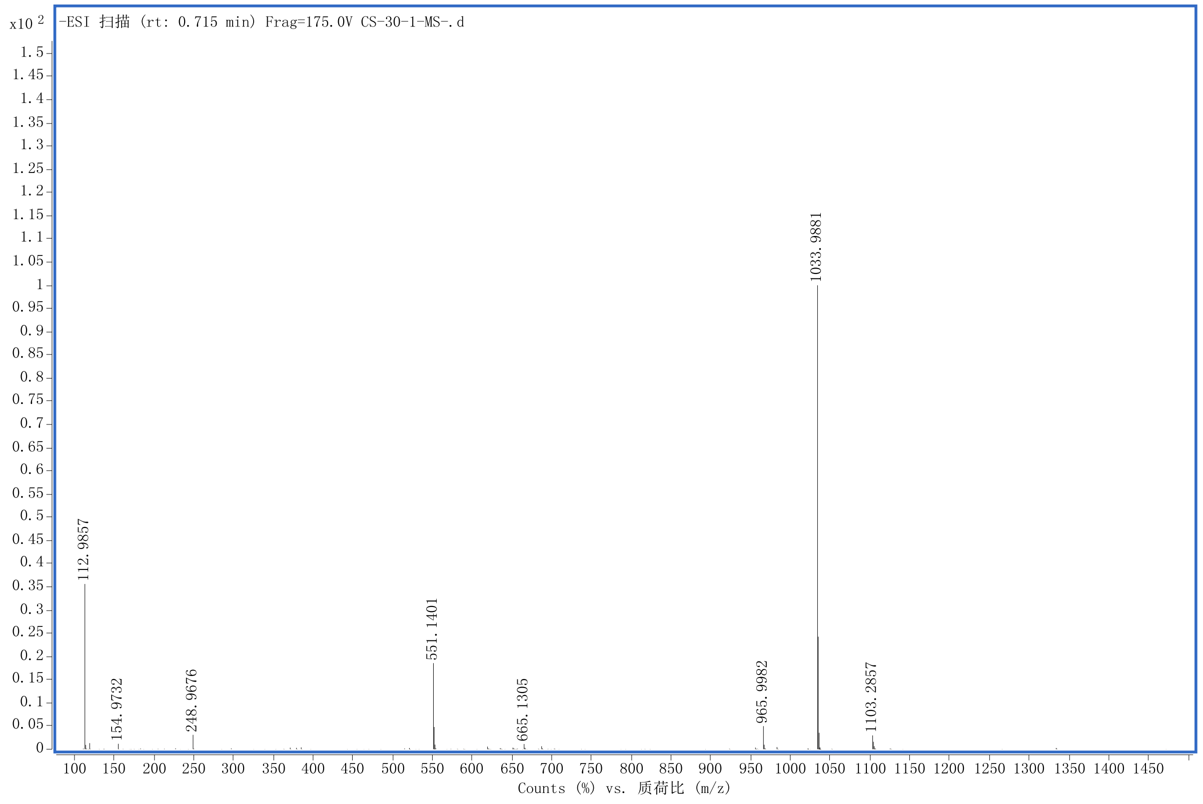

Figure S20. ^{1}H NMR (600 MHz, CD_3OD) spectrum of **3**

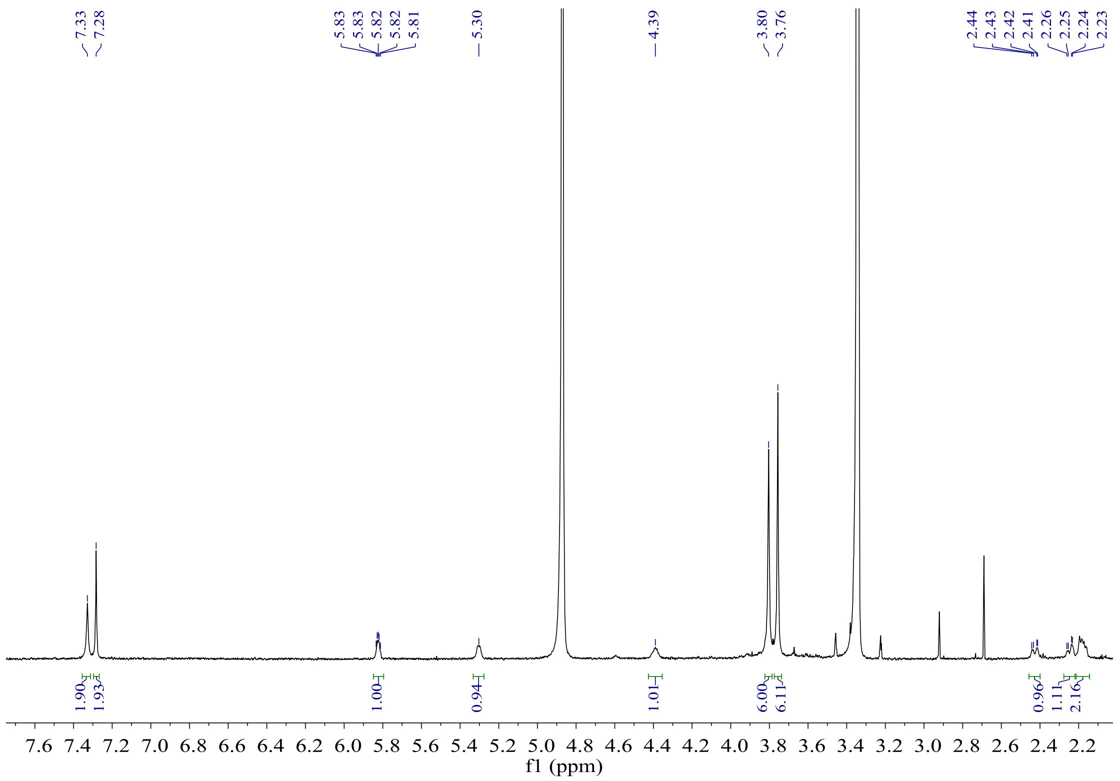

Figure S21. ^{13}C NMR (150 MHz, CD_3OD) spectrum of **3**

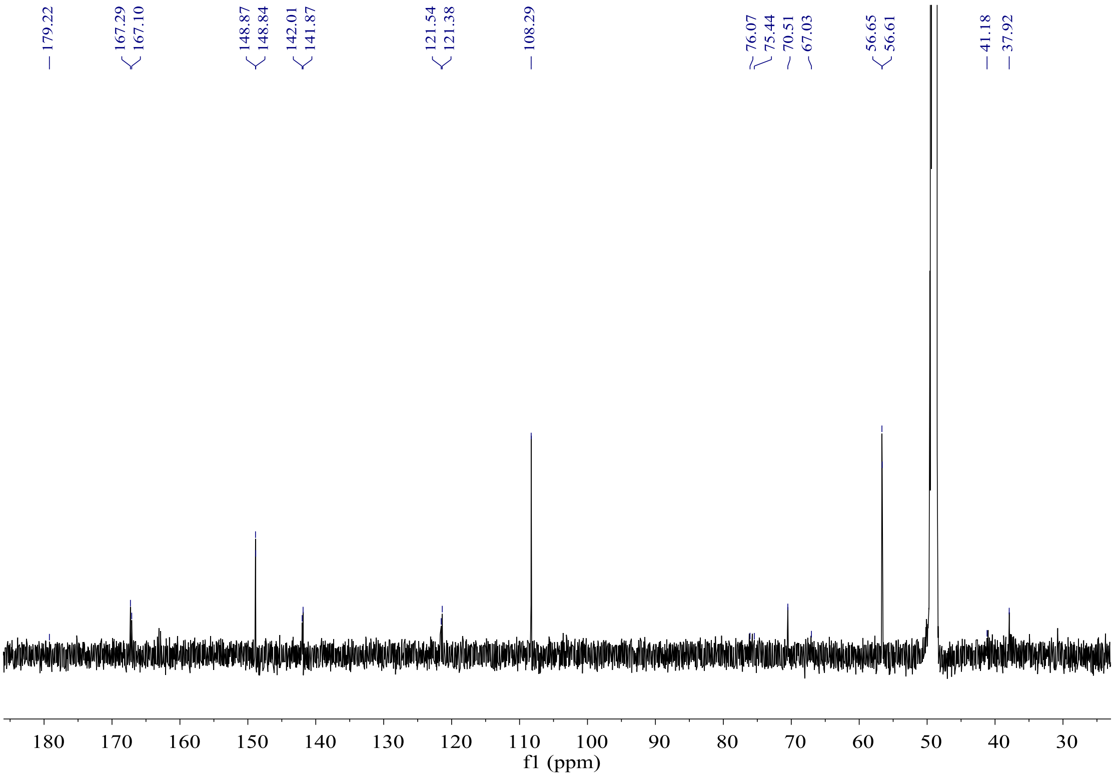

Figure S22. DEPT 135 (150 MHz, CD_3OD) spectrum of **3**

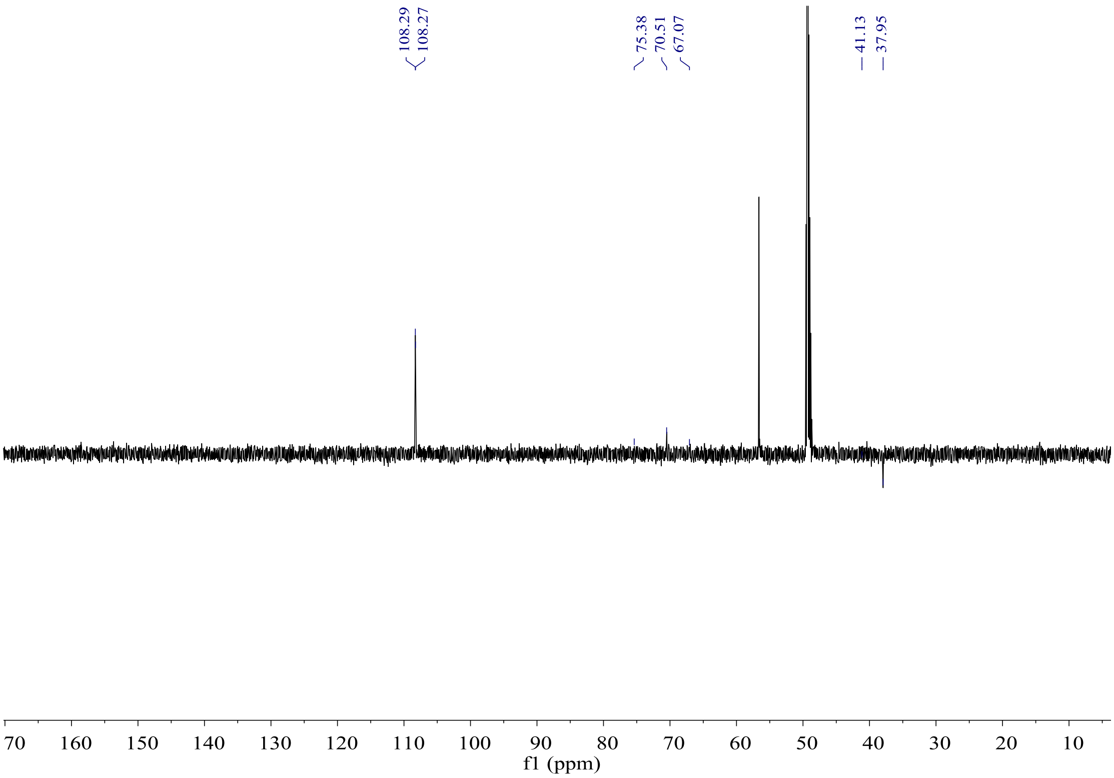

Figure S23. HSQC spectrum of **3** in CD_3OD

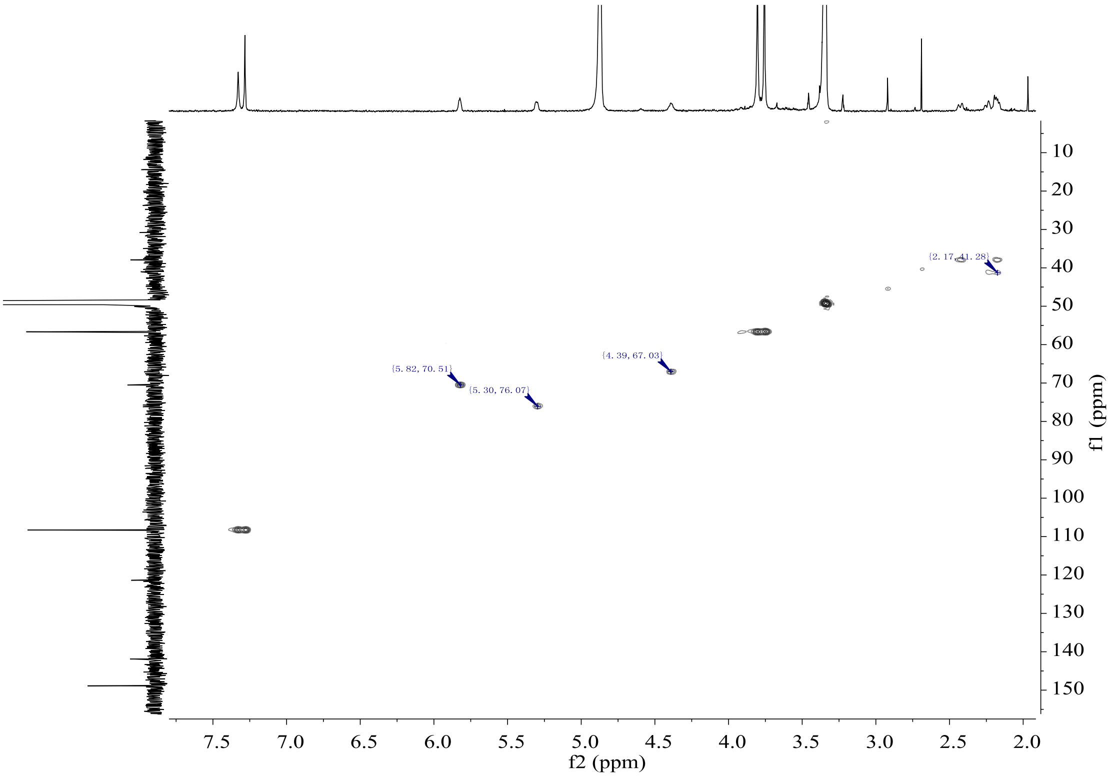

Figure S24. ^{1}H-^{1}H COSY spectrum of **30** in CD_3OD

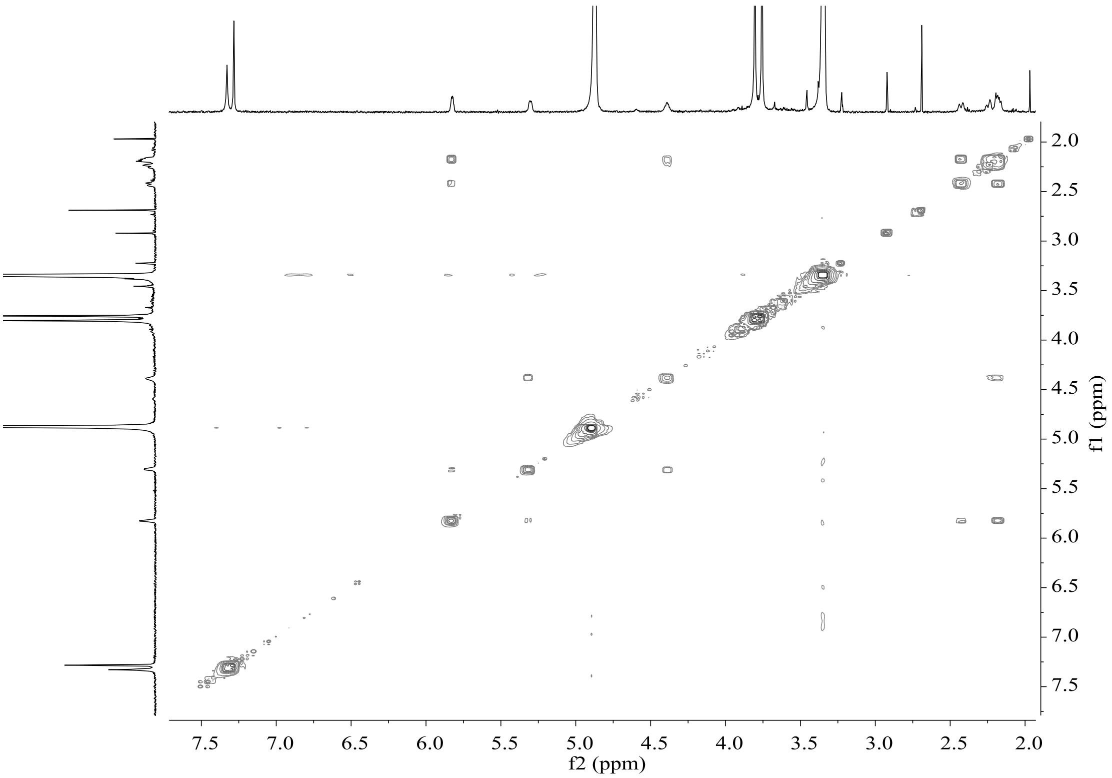

Figure S25. HMBC spectrum of **3** in CD_3OD

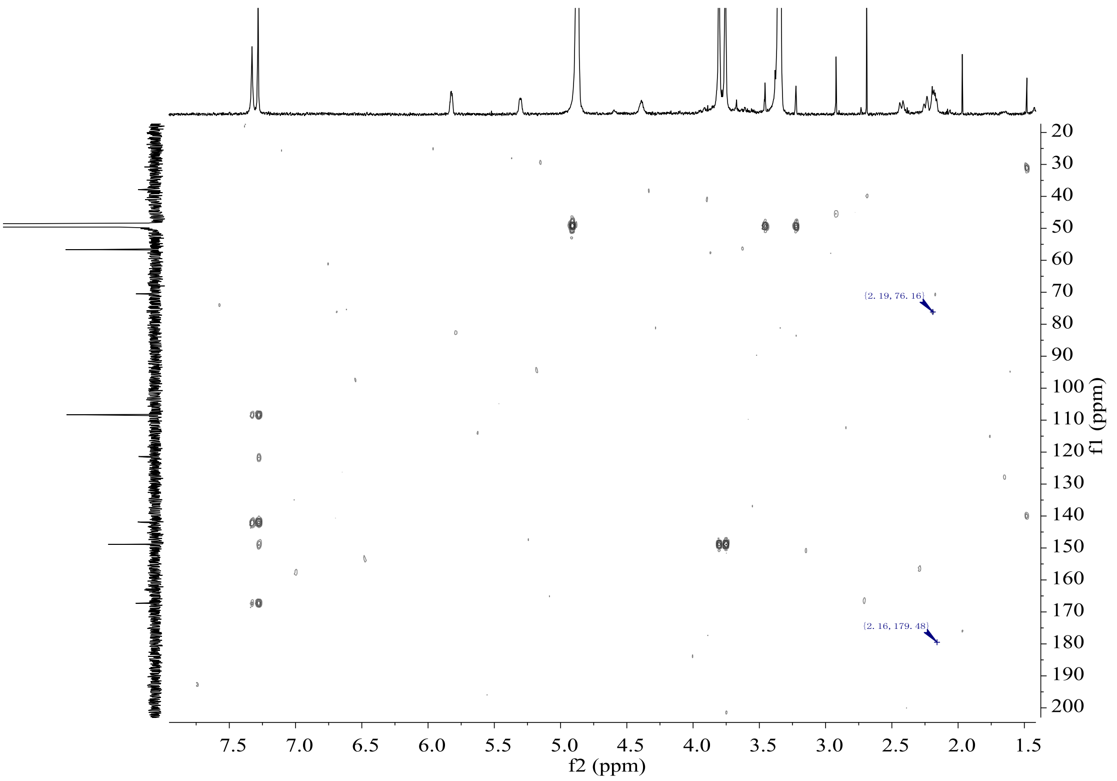

Figure S26. NOESY spectrum of **3** in CD_3OD

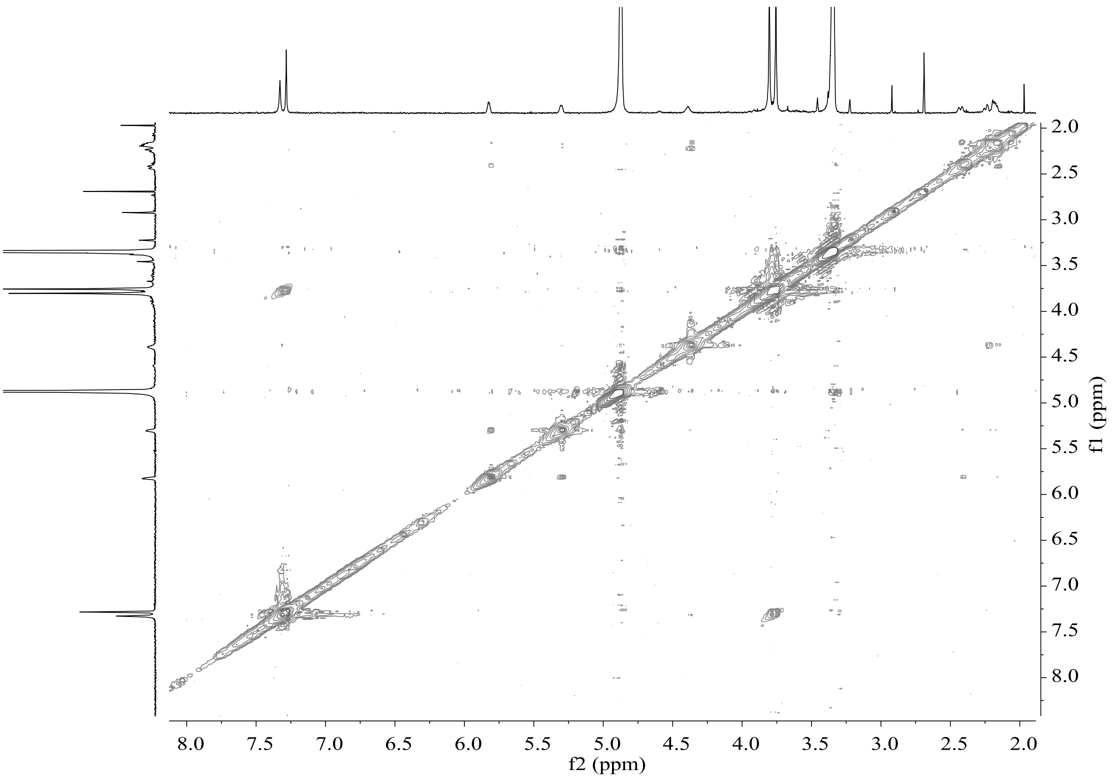

Figure S27. UV spectrum of **3** in CD_3OD

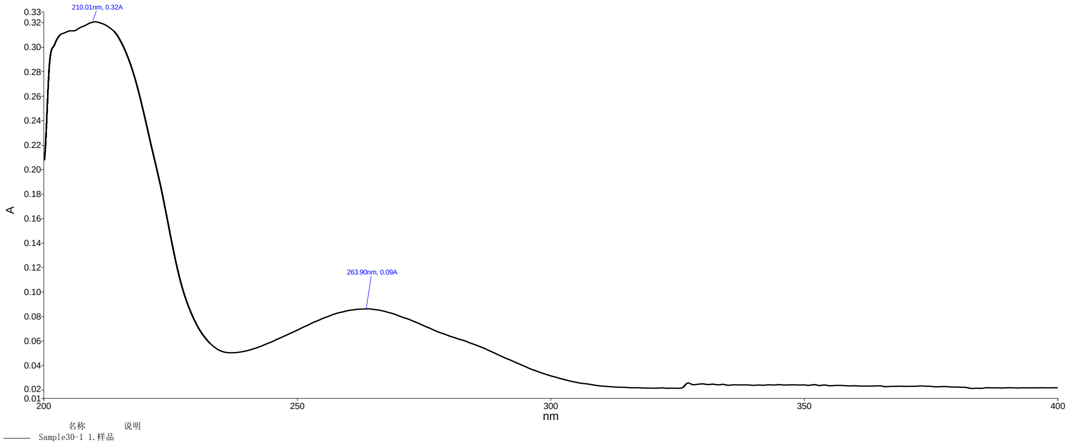

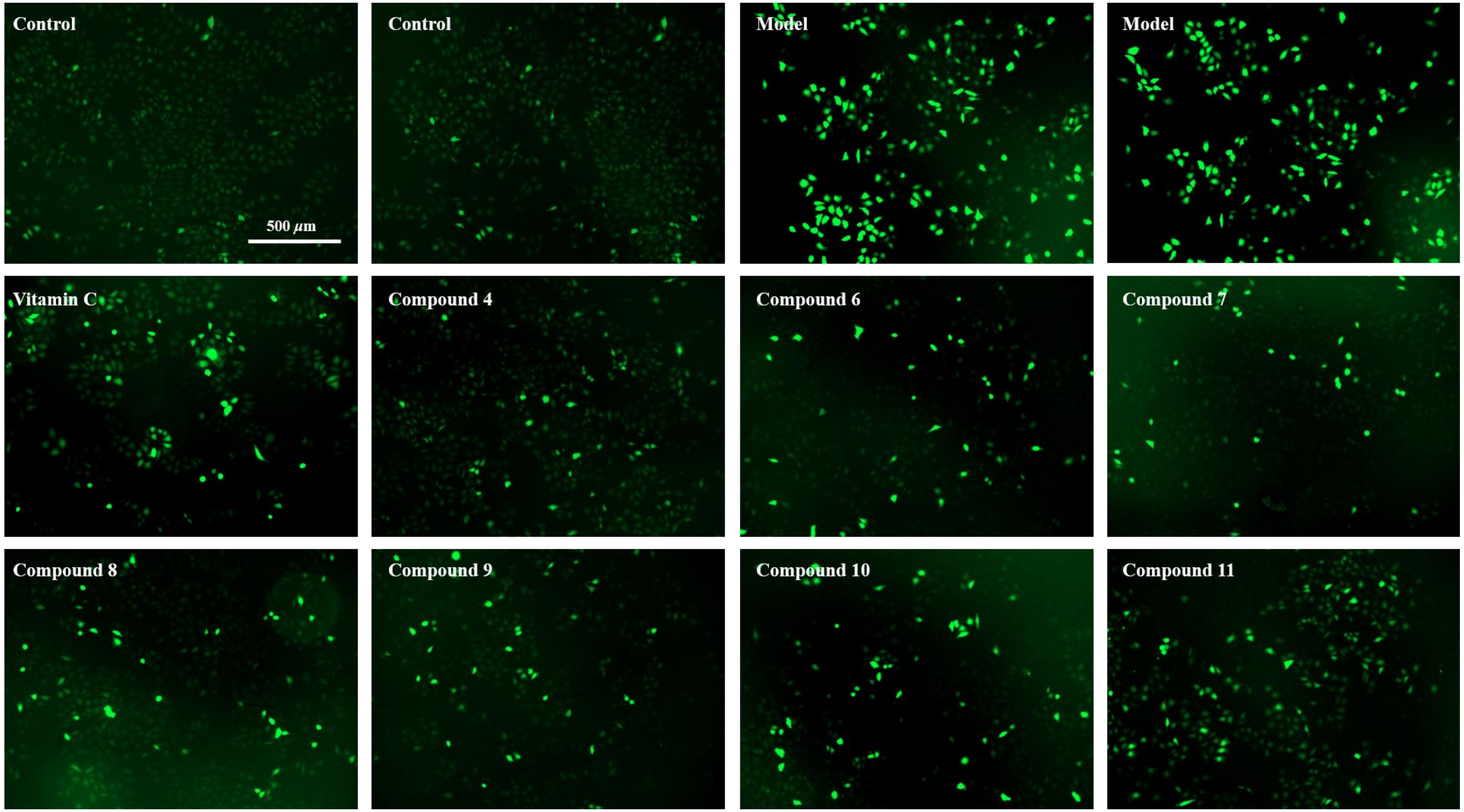

Figure S28. ROS production of compounds **4**, **6−11** and positive control vitamin C at 5 μM in H_2O_2-induced L02 cell injury model shown in fluorescence images photographed by cell imaging multi-mode reader (Cytation 1, BioTek; scale bar: 500 μm, 4 ×)
